# Genome-wide identification of SSR and SNP markers from the non-heading Chinese cabbage for comparative genomic analyses

**DOI:** 10.1186/s12864-015-1534-0

**Published:** 2015-04-20

**Authors:** Xiaoming Song, Tingting Ge, Ying Li, Xilin Hou

**Affiliations:** State Key Laboratory of Crop Genetics and Germplasm Enhancement/Key Laboratory of Biology and Germplasm Enhancement of Horticultural Crops in East China, Ministry of Agriculture, Nanjing Agricultural University, Nanjing, 210095 China

**Keywords:** Non-heading Chinese cabbage, Comparative genomic analysis, SSR, SNP

## Abstract

**Background:**

Non-heading Chinese cabbage (NHCC), belonging to *Brassica*, is an important leaf vegetable in Asia. Although genetic analyses have been performed through conventional selection and breeding efforts, the domestication history of NHCC and the genetics underlying its morphological diversity remain unclear. Thus, the reliable molecular markers representative of the whole genome are required for molecular-assisted selection in NHCC.

**Results:**

A total of 20,836 simple sequence repeats (SSRs) were detected in NHCC, containing repeat types from mononucleotide to nonanucleotide. The average density was 62.93 SSRs/Mb. In gene regions, 5,435 SSRs were identified in 4,569 genes. A total of 5,008 primer pairs were designed, and 74 were randomly selected for validation. Among these, 60 (81.08%) were polymorphic in 18 *Cruciferae*. The number of polymorphic bands ranged from two to five, with an average of 2.70 for each primer. The average values of the polymorphism information content, observed heterozygosity, Hardy-Weinberg equilibrium, and Shannon’s information index were 0.2970, 0.4136, 0.5706, and 0.5885, respectively. Four clusters were classified according to the unweighted pair-group method with arithmetic average cluster analysis of 18 genotypes. In addition, a total of 1,228,979 single nucleotide polymorphisms (SNPs) were identified in the NHCC through a comparison with the genome of Chinese cabbage, and the average SNP density in the whole genome was 4.33/Kb. The number of SNPs ranged from 341,939 to 591,586 in the 10 accessions, and the average heterozygous SNPs ratio was ~42.53%. All analyses showed these markers were high quality and reliable. Therefore, they could be used in the construction of a linkage map and for genetic diversity studies for NHCC in future.

**Conclusions:**

This is the first systematic and comprehensive analysis and identification of SSRs in NHCC and 17 species. The development of a large number of SNP and SSR markers was successfully achieved for NHCC. These novel markers are valuable for constructing genetic linkage maps, comparative genome analysis, quantitative trait locus (QTL) mapping, genome-wide association studies, and marker-assisted selection in NHCC breeding system research.

**Electronic supplementary material:**

The online version of this article (doi:10.1186/s12864-015-1534-0) contains supplementary material, which is available to authorized users.

## Background

Non-heading Chinese cabbage (*Brassica rapa* ssp. *chinensis*, 2n = 2x = 20) is a species belonging to the *Brassica* genus of *Cruciferae* family, which contains 338 genera and over 3,700 species, including the model plant *Arabidopsis* [1]. In 2012, the production of *Brassica* vegetables reached 70.10 million tons worldwide (http://faostat.fao.org). The six widely cultivated *Brassica* are described by the classical and famous “U’s triangle”, which includes three diploid species, *B. rapa* (A genome, 2n = 20), *Brassica nigra* (B genome, 2n = 16), *Brassica oleracea* (C genome, 2n = 18), and three allopolyploid species, *Brassica juncea* (AB genome, 2n = 36), *Brassica napus* (AC genome, 2n = 38) and *Brassica carinata* (BC genome, 2n = 34) [2,3]. *Brassica rapa* contains several subspecies such as Chinese cabbage (*B. rapa* ssp. *pekinensis*), NHCC and turnip (*B. rapa* ssp. *rapa*) [4]. According to the main cultivation specialties, biological properties, and morphological characteristics, NHCC are classified into five varieties, including Pak-choi, Wutatsai, Flowering Chinese cabbage, Taitsai, and Tillering cabbage [5]. NHCC is widely used as a vegetable crop because of the strong adaptability, short growth period, good quality, unique flavor, and rich nutrition. Thus, it is widely cultivated in Southeast Asia, Japan, USA, and Europe, and is gradually becoming an important vegetable worldwide.

The development of molecular markers for the detection and exploitation of DNA polymorphisms is a significant application in the field of molecular genetics. The detection and analysis of genetic variation can help us to understand the molecular basis of various biological phenomena [6,7]. Since the advent of restriction fragment length polymorphism (RFLP) markers, a range of other markers, such as random amplified polymorphism DNA (RAPD), amplified fragment length polymorphism (AFLP), sequence tag sites (STSs), SNPs, and SSRs, have been introduced during the 20th century to fulfill various demands of breeders [8,9].

Assigning molecular markers to linkage groups and constructing genetic maps is an important step for analyzing the genome of species. These linkage maps have been used for marker-assisted breeding, map-based cloning strategies, genome organization and comparative genomics of important species, and the dissection of quantitative traits [10-12]. Polymerase chain reaction-based markers have been widely used in the construction of genetic linkage maps for *B. oleracea* [13,14], *B. nigra* [15], *B. juncea* [16], and *B. napus* [17,18]. A number of genetic linkage maps based on a range of markers, including RFLP, RAPD, SSR, and AFLP, have been constructed for Chinese cabbage [3,19,20]. However, there are few linkage maps for NHCC [21].

SSR and SNP markers are distributed throughout the genome, and they gradually became preferred markers for many applications in genetics and genomics [22-24]. They are suitable for the fine mapping of genes and association studies, which aim at identifying alleles potentially affecting important agronomic traits [25,26]. However, without large numbers of SSR and SNP markers, such studies have not been available in most crop species. Currently, with the development of next-generation sequencing, it is feasible to develop a large number of SSR and SNP markers. Developing a large set of SSR and SNP markers will facilitate the fine mapping of QTLs, improve the identification and exploitation of genes affecting important traits, and enable selective breeding through genomic selection [27,28].

NHCC, with rich diverse germplasms, originated from China. Given its important economic value and its close relationship to *A. thaliana* and Chinese cabbage, 10 NHCC accessions were re-sequenced. Additionally, the representative accession, NHCC001, has been assembled recently. Here, we report, for the first time, a survey of whole genome sequences to develop a large number of SSR and SSR markers. These markers enhance the density of the existing genetic NHCC maps, which could also be a useful source for high-throughput QTL mapping and marker-assisted NHCC improvement. Furthermore, breeders could introduce beneficial genes, improving genetic diversity, using these markers for marker-assisted selection.

## Results

### The development of SSRs in NHCC and a comparative analysis with 17 species

We analyzed the distribution of perfect microsatellites with ≥ 3 repeat units, and a minimum total length of 18 bp in ~331.1 Mb of the NHCC genome. All SSRs identified in this study have been submitted to the nhccdata website (http://nhccdata.njau.edu.cn/). The content of perfect microsatellites in the genomic sequences of NHCC and 17 other species were identified. A total of 20,836 SSRs with repeats were detected in the NHCC genome, which translated to an overall density across the genome of 62.93 SSRs/Mb. Surprisingly, NHCC had a higher microsatellite density than sorghum (56.00 SSR/Mb), potato (55.78 SSR/Mb), and maize (24.85 SSR/Mb). However, its microsatellite density was far less than watermelon (217.96 SSR/Mb), moss (241.08 SSR/Mb), and volvox (377.15 SSR/Mb) (Table 1, Additional file 1: Table S1).

**Table 1:** **Summary of different SSR repeats in genomic sequences of non-heading Chinese cabbage and selected plant species**

**Species**	**Repeat type**	**Mono-**	**Di-**	**Tri-**	**Tetra-**	**Penta-**	**Hexa-**	**Hepta-**	**Octa-**	**Nona-**	**Total**	**Genome (Mb)**
*B.* rapa.ssp.*chinensis*	Count	4,975	8,296	3,797	754	1,125	589	982	197	121	20,836	331.09
	Density (SSR/Mb)	15.03	25.06	11.47	2.28	3.40	1.78	2.97	0.60	0.37	62.93	
*Arabidopsis thaliana*	Count	2,430	3,383	2,350	169	351	178	691	82	39	9,673	119.67
	Density (SSR/Mb)	20.31	28.27	19.64	1.41	2.93	1.49	5.77	0.69	0.33	80.83	
*B.* rapa.ssp.*pekinensis*	Count	4,665	11,431	3,873	962	1,085	607	1,080	256	136	24,095	283.84
	Density (SSR/Mb)	16.44	40.27	13.65	3.39	3.82	2.14	3.80	0.90	0.48	84.89	
*Cucumis sativus*	Count	1,415	11,305	5,573	1,521	1,758	1,148	2,337	764	265	26,086	203.06
	Density (SSR/Mb)	6.97	55.67	27.45	7.49	8.66	5.65	11.51	3.76	1.31	128.46	
*Glycine max*	Count	8,247	59,923	19,053	3,954	4,686	1,649	6,482	562	297	104,853	973.34
	Density (SSR/Mb)	8.47	61.56	19.57	4.06	4.81	1.69	6.66	0.58	0.31	107.72	
*Medicago truncatula*	Count	11,367	13,030	5,701	1,442	1,882	958	1,121	235	67	35,803	418.58
	Density (SSR/Mb)	27.16	31.13	13.62	3.44	4.50	2.29	2.68	0.56	0.16	85.53	
*Oryza sativa*	Count	1,671	13,818	11,948	2,579	2,963	1,398	968	321	91	35,757	374.47
	Density (SSR/Mb)	4.46	36.90	31.91	6.89	7.91	3.73	2.58	0.86	0.24	95.49	
*Physcomitrella patens*	Count	19,402	60,933	8,940	9,365	6,910	1,822	5,653	2,027	664	115,716	479.99
	Density (SSR/Mb)	40.42	126.95	18.63	19.51	14.40	3.80	11.78	4.22	1.38	241.08	
*Populus trichocarpa*	Count	3,170	18,071	9,127	2,048	2,562	1,553	1,845	277	91	38,744	265.70
	Density (SSR/Mb)	11.93	68.01	34.35	7.71	9.64	5.84	6.94	1.04	0.34	145.82	
*Sorghum bicolor*	Count	2,251	14,331	11,895	5,368	3,162	2,648	1,397	170	134	41,356	738.54
	Density (SSR/Mb)	3.05	19.40	16.11	7.27	4.28	3.59	1.89	0.23	0.18	56.00	
*Solanum lycopersicum*	Count	904	32,484	18,053	3,272	1,761	1,581	1,919	319	231	60,524	781.67
	Density (SSR/Mb)	1.16	41.56	23.10	4.19	2.25	2.02	2.46	0.41	0.30	77.43	
*Solanum tuberosum*	Count	4,575	14,709	9,962	1,514	2,700	1,705	3,352	566	293	39,376	705.88
	Density (SSR/Mb)	6.48	20.84	14.11	2.14	3.83	2.42	4.75	0.80	0.42	55.78	
*Volvox carteri*	Count	58	6,899	19,469	10,016	3,394	3,550	1,509	2,652	1,920	49,467	131.16
	Density (SSR/Mb)	0.44	52.60	148.44	76.36	25.88	27.07	11.51	20.22	14.64	377.15	
*Vitis vinifera*	Count	17,217	27,418	16,025	6,435	4,863	2,076	6,032	1,378	312	81,756	486.20
	Density (SSR/Mb)	35.41	56.39	32.96	13.24	10.00	4.27	12.41	2.83	0.64	168.15	
*Zea mays*	Count	4,443	17,959	15,080	3,316	4,861	2,682	2,153	429	424	51,347	2,066.43
	Density (SSR/Mb)	2.15	8.69	7.30	1.60	2.35	1.30	1.04	0.21	0.21	24.85	
*Citrus sinensis*	Count	5,996	11,723	9,759	2,769	2,823	1,152	2,095	671	179	37,167	327.94
	Density (SSR/Mb)	18.28	35.75	29.76	8.44	8.61	3.51	6.39	2.05	0.55	113.33	
*Musa acuminata*	Count	1,599	35,516	10,781	2,214	1,490	1,169	960	487	60	54,276	437.28
	Density (SSR/Mb)	3.66	81.22	24.65	5.06	3.41	2.67	2.20	1.11	0.14	124.12	
*Citrullus lanatus*	Count	32,837	13,474	13,646	5,465	4,205	2,082	4,059	1,291	373	77,432	355.25
	Density (SSR/Mb)	92.43	37.93	38.41	15.38	11.84	5.86	11.43	3.63	1.05	217.96	

Dinucleotides were the most common SSR type in NHCC genomic sequences, representing 39.82% of all SSRs, followed by mono- (23.88%) and trinucleotides (18.22%). Octa- and nonanucleotides were the least frequent repeat types, together representing less than 2% of the total SSRs (Table 2). The distribution of SSR types in NHCC was most similar to those of Chinese cabbage and *Arabidopsis*, which had comparable relative and absolute frequencies for each SSR type, and it was at least similar to the distribution in volvox and watermelon, for which trinucleotides were by far the most frequent repeat type. There were many more dinucleotides and trinucleotides than that in moss and volvox, respectively (Additional file 2: Figure S1, Additional file 1: Table S2).

**Table 2:** **Summary of different SSR repeats in genomic and gene sequences of non-heading Chinese cabbage**

**Repeat type**	**Genome SSR**	**Percentage (%) (SSR/Total genome)**	**Gene SSR**	**Percentage (%) (SSR/Total gene)**	**SSR length**	**Average repeat times**
Mononucleotide	4,975	23.88	904	16.63	101,382	20.38
Dinucleotide	8,296	39.82	1,532	28.19	288,420	17.38
Trinucleotide	3,797	18.22	2,091	38.47	80,829	7.10
Tetranucleotide	754	3.62	233	4.29	19,772	6.56
Pentanucleotide	1,125	5.40	267	4.91	22,745	4.04
Hexanucleotide	589	2.83	210	3.86	17,214	4.87
Heptanucleotide	982	4.71	147	2.70	22,988	3.34
Octanucleotide	197	0.95	26	0.48	4,408	2.80
Nonanucleotide	121	0.58	25	0.46	3,222	2.96

**Figure 1:**
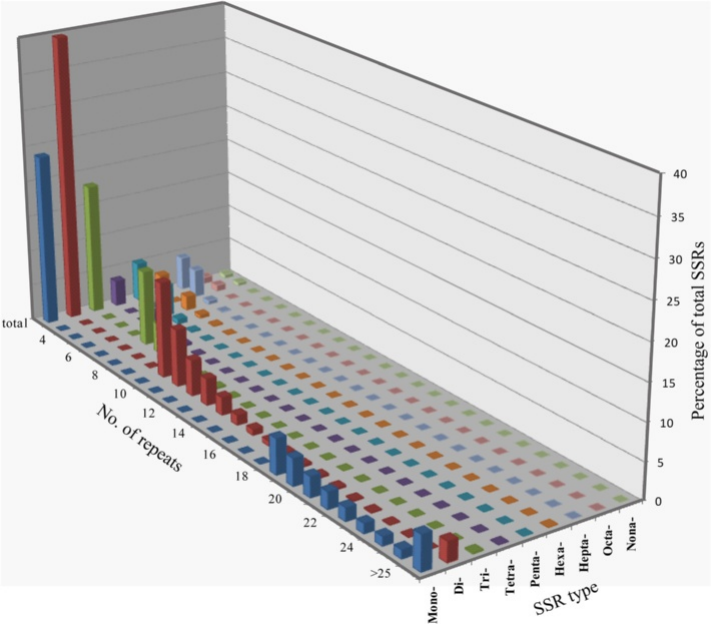
Relative frequency of SSR types, characterized by the number of repeats in the non-heading Chinese cabbage. The graph was based on a total of 20,836 SSRs detected in a 331-Mb, non-redundant genomic DNA sequence of the NHCC genome.

We examined the distribution of NHCC microsatellites with regard to the number of repeat units (Figure 1, Additional file 1: Table S3). For all SSR classes, the microsatellite frequency decreased as the number of repeat units increased. However, the rate of this change was more gradual for mononucleotides and dinucleotide than for longer repeat types, with pentanucleotide (from 919 to 154) to nonanucleotide (from 104 to 10) showing the most dramatic frequency reduction. Moreover, the total length of dinucleotide sequences was much larger than the other repetitive sequences, with a total length of 288.42 Kb. On the one hand, the mean number of repeat units in the dinucleotides (17.38) was over twice as high as the number of repeat units in the trinucleotides (7.10), and it was four times higher than in penta- to nonanucleotides (4.04–2.96) (Table 2). On the other hand, the dinucleotide repeats (25.06 SSR/Mb) occurred more frequently than other dinucleotides in the NHCC. Therefore, the dinucleotide repeats had a greater contribution to the genome fraction occupied by SSRs than other dinucleotide (Table 1).

Of the 20,836 identified SSR markers, 822 were not anchored on chromosomes. In addition, the number of SSR markers was different on each chromosome. The density of SSRs ranged from 67.25 to 73.53 across the chromosomes. The most SSR markers (2,996, 14.38%) occurred on chromosome 9, while the least were on chromosome 4 (1,337, 6.42%) (Additional file 1: Table S4). A total of 1,685 types of repeat motifs were detected in NHCC genomic SSR. The most type was the A/T (4,185, accounting for 20.09%), followed by AG/CT (4,107, 19.71%), AT/TA (3,700, 17.76%), and AAG/CTT (1,281, 6.15%), which was similar to other *Cruciferous* species. The remaining types had repeat ratios of less than 4%, and the CG/GC repeat motif was not found among the NHCC genomic SSRs (Additional file 1: Table S5).

### The characteristics of SSR markers in NHCC genes and a functional analysis

A total of 5,435 SSRs were identified in NHCC genes, accounting for 26.08% of the total genomic SSRs. Trinucleotides were the most common SSR type, representing 38.47% (2,091) of all genic SSRs. Even though dinucleotides are the most common type in the genome, trinucleotides may be more common in the gene regions because they do not cause gene translational changes. They are followed by dinucleotide (1,532, 28.19%) and mononucleotides (904, 16.63%) (Table 2). In total, 611 repeat motifs were identified in NHCC genes, and the most type was AG/CT (783, 14.41%), followed by A/T (763, 14.13%), AAG/CTT (631, 11.61%), and AT/TA (582, 10.71%). The other repeat motifs occurred at rates of less than 10.00%, which was similar to their rates in the genome (Additional file 1: Table S6).

These SSR markers were located in 4,569 genes, accounting for 10.97% of the total number of genes, and 708 genes contained several SSR markers. The functions of 3,141 genes containing SSRs were divided into three classes, cellular location, molecular function, and biological process. They were further subdivided into 38 functional subsets. The greatest number of genes was associated with the binding factors (2,245, 71.47%), followed by the genes involved in metabolic processes, catalytic activities, and cellular processes. This was similar to the classification of the 3,036 genes containing non-synonymous SNPs (Additional file 3: Figure S2).

**Figure 2:**
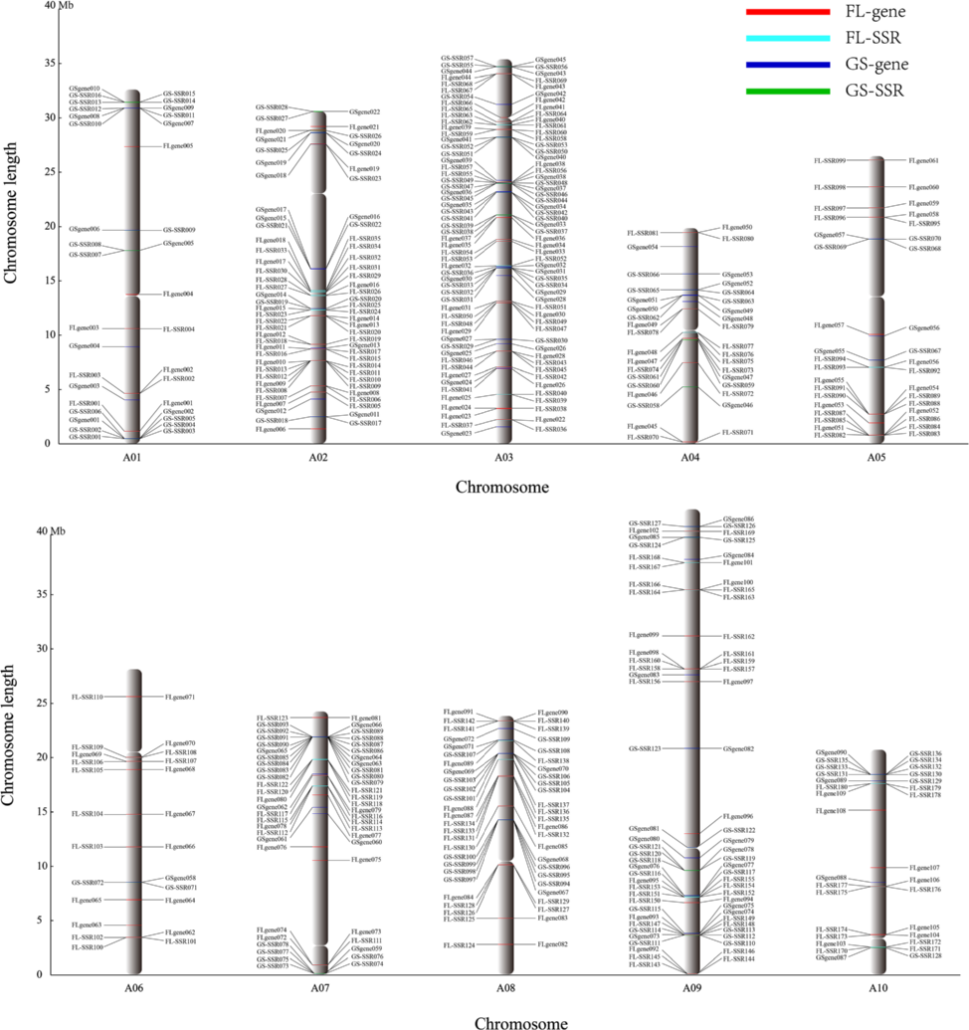
Chromosomal locations of non-heading Chinese cabbage flower (FL) and glucosinolate (GS) genes with related SSR markers (<40 Kb). In total, 180 (FL-SSR) and 136 (GS-SSR) SSRs were depicted on the 10 NHCC chromosomes. Scale is in megabases (Mb).

SSRs located near important functional genes, such as flower genes and glucosinolate genes, were also identified. Most plants undergo a major physiological change from vegetative to reproductive development before flowering. The formation of flowers is a prerequisite for successful sexual reproduction, and fruits of angiosperm flowers are a staple of human and livestock diets [29]. Glucosinolates are a category of amino acid-derived secondary metabolites found in the *Cruciferae* family. Glucosinolates and their degradation products play important roles in pathogen and insect interactions, especially in human health [30]. Based on their importance, we identified these genes and their related SSR markers in NHCC. In our analysis, 110 and 93 genes showed high homology (>90%) to the Chinese cabbage flower genes and glucosinolate genes, respectively. Finally, 180 and 136 SSRs were found in the vicinity of (<40 Kb) 86 flower genes and 62 glucosinolate genes, respectively. Interestingly, the number of these genes and related SSRs on chromosome 9 was more than on each of the other nine chromosomes. These markers will be useful for marker-assisted selection breeding in the future (Figure 2, Additional file 1: Table S7).

### The abundance and length frequency analyses of SSR repeat motifs

We conducted a detailed analysis of individual repeat motifs for each type of SSR found in the genomic sequences of NHCC and the other 17 species. The results showed that A/T (84.12%), AG/CT (49.51%), AAG/CTT (33.74%), AAAT/ATTT (27.06%), AAAAT/ATTTT (20.62%), AAAAAT/ATTTTT (9.51%), AAACCCT/AGGGTTT (12.73%), AAAAAAAT/ATTTTTTT (10.66%), and AAAATAAAT/ATTTATTTT (8.26%) were the most frequent motifs from mono- to nonanucleotides in the NHCC genome. A/T repeats were not only the predominant mononucleotide, but they were also the most frequent motif in the entire genome, accounting for 20.09% of the total SSRs, followed by AG/CT (19.71%) and AT/AT (17.76%) repeats. These three repeat types were more than half of the total SSRs in NHCC genomic sequences (Additional file 1: Table S5). In addition, the motif density was also calculated in the other 17 species for a comparative analysis. The results showed that the density of A/T repeats was higher than C/G repeats in most examined species (14/18). For dinucleotides, all species had a relative low density (0–0.17) of CG/CG repeats. The number of AT/AT repeats was higher than other dinucleotides in 17 species. However, AG/CT repeats (12.40) were slightly more abundant than AT/AT repeats (11.18) in NHCC. Surprisingly, the density of AC/GT repeats (44.64) was far greater than of other dinucleotides in volvox. The density of AAG/CTT repeats was greater than other trinucleotide in *Cruciferous* (*Arabidopsis*, Chinese cabbage and NHCC), which was different from the other examined species. Most species had a higher density of AAT/ATT repeats than other trinucleotide repeats. However, the density of CCG/CGG repeats was higher than other trinucleotides in rice and volvox. In NHCC, as well as in most of other species examined, the frequencies of different tetranucleotides revealed that repeats of AAAT/ATTT were most common, whereas ACAT/ATGT (36.96) and AGAT/ATCT (1.64) repeats predominated in volvox and rice, respectively. Conversely, the GC-rich motifs were of relatively lower densities in most species analyzed, such as CCCG/CGGG and CCGG/CCGG. However, the opposite distribution was observed in volvox (Additional file 1: Table S2, Additional file 4: Figure S3a–c).

### The polymorphism analysis of SSR markers among 18 *Cruciferae* accessions

A total of 5,008 (92.14%) SSR primer pairs were designed from the 5,435 SSRs in the gene sequences. Of these, 74 primer pairs were selected for validation by SSR loci amplification, and 63 produced a reproducible and clear amplicon of the expected size. The product sizes ranged from 101 to 280 bp. A total of 60 (81.08%) were polymorphic among the 18 analyzed species of *Cruciferae*, including one *Arabidopsis*, two broccoli, one Chinese cabbage, and 14 NHCC accessions (Additional file 1: Table S8, Table S9).

**Figure 3:**
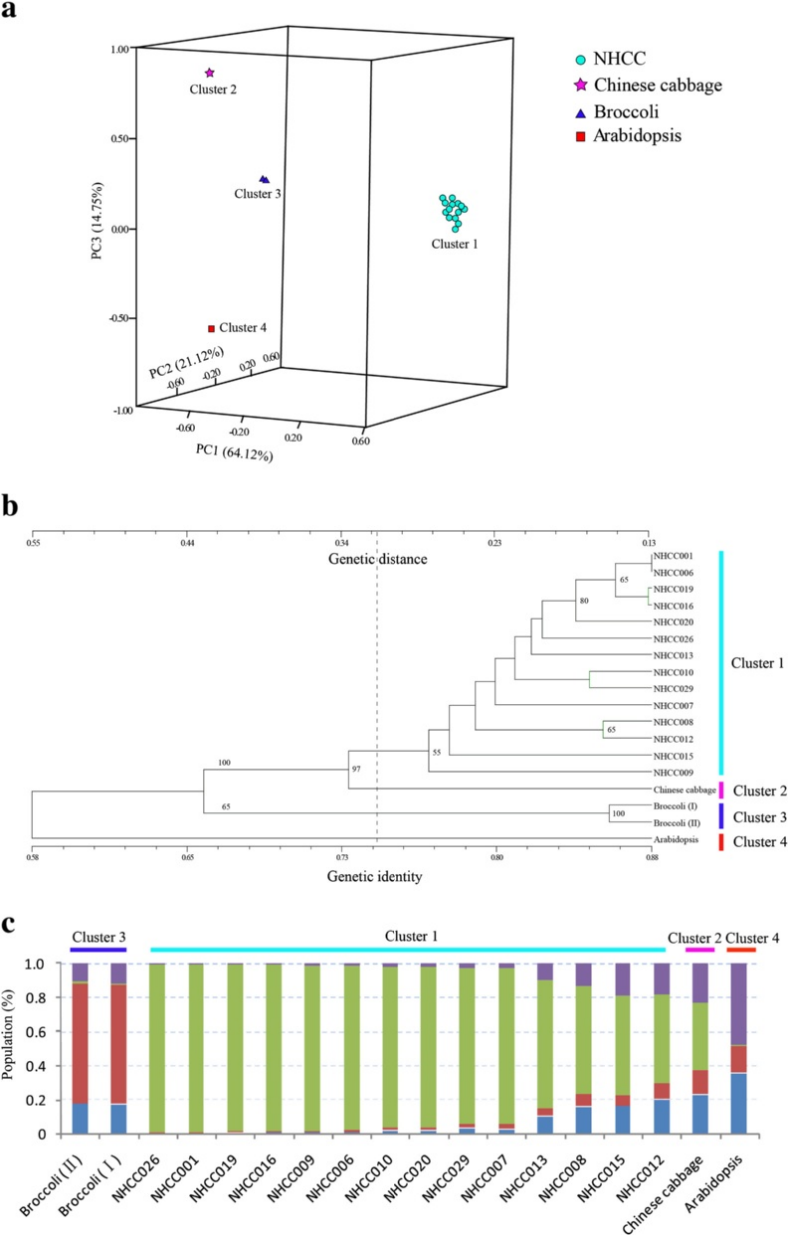
A cluster analyses of 18 *Cruciferae* accessions. **(a)** Plot of the three principal components from a principal components analysis of SSR variation among 18 genotypes of *Cruciferae*. Green circles represent non-heading Chinese cabbage accessions; pink pentagrams represent Chinese cabbage; blue triangles represent broccoli accessions; and red squares represent *Arabidopsis*. **(b)** Dendrogram for 18 *Cruciferae* accessions derived from a UPGMA cluster analysis based on 60 SSR markers. Four clusters were obtained according to the genetic identity (~75%). Cluster 1 indicated NHCC accessions; Cluster 2 indicated Chinese cabbage; Cluster 3 indicated broccoli accessions; and Cluster 4 indicated *Arabidopsis*. The numbers were bootstrap values based on 1,000 iterations. Only bootstrap values larger than 50 were indicated. **(c)** Bayesian clustering (STRUCTURE, K = 4) of 18 *Cruciferae* accessions.

A total of 162 polymorphic bands were produced by 60 primer pairs in the 18 accessions. The number of polymorphic bands ranged from two to five, with an average of 2.70 for each primer. The major allele frequency at each locus ranged from 0.4667 to 0.9722. The polymorphism information content (PIC) at each locus ranged from 0.0526 to 0.5802, with an average of 0.2970/loci. The expected heterozygosity ranged from 0.0556 to 0.6506, and the observed heterozygosity ranged from 0.0000 to 1.0000. Although a limited number of SSR primers were used in this experiment, they produced rich polymorphic bands in the 18 *Cruciferae* accessions. The gene flow estimated from F-statistics was from 0.0000 to 4.5000. A total of 13 SSR primers showed significant deviations from the Hardy–Weinberg equilibrium (P_HW_ < 0.05). Nei’s genetic identity ranged from 0.5165 to 0.8799, and the genetic distance ranged from 0.1280 to 0.6439. Shannon’s information index ranged from 0.1269 to 1.2203, with an average of 0.5885 (Table 3, Additional file 1: Table S10). These results indicated that a large amount of genetic diversity in the 18 *Cruciferae* had been assessed. The dendrogram showed that they fell into four distinct clusters. Cluster 1 was comprised of 14 NHCC accessions, including five varieties of NHCC. Chinese cabbage belonged to Cluster 2, which had a close relationship with the Taitsai variety of NHCC. Clusters 3 and 4 contained broccoli and *Arabidopsis*, respectively. The principal component analysis (PCA) and population structure analysis corroborated this classification (Figure 3).

**Table 3:** **Information of SSR loci following amplification from 18** ***Cruciferae*** **accessions**

**Locus**	**MAF**	**Gn**	**Na**	**Ne**	**I**	**Ho**	**He**	**Fst**	**Nm**	**Gd**	**PIC**	**P**_**HW**_
BrcSSR01	0.5357	3	2	1.9898	0.6906	0.0714	0.5159	0.9601	0.0104	0.4974	0.3737	0.0030
BrcSSR02	0.9375	3	3	1.1353	0.2771	0.1250	0.1230	0.8173	0.0559	0.1191	0.1157	1.0000
BrcSSR03	0.7000	2	2	1.7241	0.6109	0.6000	0.4345	0.5814	0.1800	0.4200	0.3318	0.2230
BrcSSR04	0.7308	2	2	1.6488	0.5825	0.5385	0.4092	0.7156	0.0994	0.3935	0.3161	0.4870
BrcSSR05	0.8235	3	3	1.4378	0.5783	0.3529	0.3137	0.5610	0.1957	0.3045	0.2809	1.0000
BrcSSR06	0.9063	2	2	1.2047	0.3111	0.1875	0.1754	0.7578	0.0799	0.1699	0.1555	1.0000
BrcSSR07	0.6111	3	3	1.9817	0.7683	0.7778	0.5095	0.2150	0.9130	0.4954	0.3972	0.0210
BrcSSR08	0.8750	2	2	1.2800	0.3768	0.2500	0.2258	0.7097	0.1023	0.2188	0.1948	1.0000
BrcSSR09	0.6875	2	2	1.7534	0.6211	0.6250	0.4435	0.4944	0.2557	0.4297	0.3374	0.2530
BrcSSR10	0.8214	3	3	1.4465	0.5894	0.3571	0.3201	0.7613	0.0784	0.3087	0.2862	1.0000
BrcSSR11	0.8125	2	2	1.4382	0.4826	0.3750	0.3145	0.6301	0.1467	0.3047	0.2583	1.0000
BrcSSR12	0.8611	3	3	1.3252	0.4724	0.2778	0.2524	0.4340	0.3261	0.2454	0.2259	1.0000
BrcSSR13	0.8824	3	3	1.2731	0.4438	0.2353	0.2210	0.6289	0.1475	0.2145	0.2037	1.0000
BrcSSR14	0.9063	3	3	1.2104	0.3708	0.1875	0.1794	0.7600	0.0789	0.1738	0.1658	1.0000
BrcSSR15	0.5909	3	3	2.1802	0.8932	0.8182	0.5671	0.6983	0.1080	0.5413	0.4632	0.2370
BrcSSR16	0.5667	3	2	1.9651	0.6842	0.3333	0.5080	0.7852	0.0684	0.4911	0.3705	0.2940
BrcSSR17	0.8889	2	2	1.2462	0.3488	0.2222	0.2032	0.4375	0.3214	0.1975	0.1780	1.0000
BrcSSR18	0.8667	3	3	1.3120	0.4677	0.2667	0.2460	0.7639	0.0773	0.2378	0.2211	1.0000
BrcSSR19	0.6944	3	3	1.8567	0.7945	0.6111	0.4746	0.3378	0.4901	0.4614	0.4064	0.4530
BrcSSR20	0.8571	2	2	1.3243	0.4101	0.2857	0.2540	0.7955	0.0643	0.2449	0.2149	1.0000
BrcSSR21	0.5000	2	3	2.2192	0.8676	1.0000	0.5651	0.0899	2.5312	0.5494	0.4479	0.0000
BrcSSR22	0.5278	2	2	1.9938	0.6916	0.9444	0.5127	0.0526	4.5000	0.4985	0.3742	0.0010
BrcSSR23	0.5556	3	3	2.3143	0.9369	0.8889	0.6013	0.7509	0.0829	0.5679	0.4889	0.2350
BrcSSR24	0.8125	2	2	1.4382	0.4826	0.3750	0.3145	0.6301	0.1467	0.3047	0.2583	1.0000
BrcSSR25	0.9167	2	2	1.1803	0.2868	0.1667	0.1594	0.9109	0.0245	0.1528	0.1411	1.0000
BrcSSR26	0.8056	3	3	1.4761	0.5723	0.3889	0.3317	0.3971	0.3795	0.3225	0.2854	1.0000
BrcSSR27	0.8889	4	4	1.2583	0.4644	0.2222	0.2111	0.4586	0.2951	0.2052	0.1979	1.0000
BrcSSR28	0.6667	2	2	1.8000	0.6365	0.6667	0.4598	0.5477	0.2064	0.4444	0.3457	0.1120
BrcSSR29	0.5625	3	2	1.9692	0.6853	0.7500	0.5081	0.4433	0.3140	0.4922	0.3711	0.1300
BrcSSR30	0.5882	4	3	2.2403	0.9238	0.7059	0.5704	0.4462	0.3103	0.5536	0.4818	0.0440
BrcSSR31	0.5000	5	4	2.5424	1.0755	0.4667	0.6276	0.7325	0.0913	0.6067	0.5326	0.0720
BrcSSR32	0.5000	2	3	2.3226	0.9184	1.0000	0.5857	0.1220	1.8000	0.5694	0.4768	0.0000
BrcSSR33	0.9167	3	3	1.1846	0.3399	0.1667	0.1603	0.4653	0.2872	0.1559	0.1494	1.0000
BrcSSR34	0.9706	2	2	1.0606	0.1327	0.0588	0.0588	0.8252	0.0529	0.0571	0.0555	1.0000
BrcSSR35	0.9118	2	2	1.1918	0.2984	0.1765	0.1658	0.6687	0.1239	0.1609	0.1480	1.0000
BrcSSR36	0.9000	2	2	1.2195	0.3251	0.2000	0.1862	0.8065	0.0600	0.1800	0.1638	1.0000
BrcSSR37	0.9412	2	2	1.1245	0.2237	0.1176	0.1141	0.7313	0.0918	0.1107	0.1046	1.0000
BrcSSR38	0.6667	3	3	1.9059	0.7867	0.6667	0.4889	0.2987	0.5870	0.4753	0.4035	0.2370
BrcSSR39	0.9167	3	3	1.1846	0.3399	0.1667	0.1603	0.4653	0.2872	0.1559	0.1494	1.0000
BrcSSR40	0.7778	3	3	1.5844	0.6767	0.4444	0.3794	0.3975	0.3789	0.3688	0.3368	1.0000
BrcSSR41	0.9444	2	2	1.1172	0.2146	0.0000	0.1079	1.0000	0.0000	0.1049	0.0994	0.0290
BrcSSR42	0.5588	7	5	2.6514	1.2203	0.3529	0.6417	0.7488	0.0839	0.6228	0.5802	0.0020
BrcSSR43	0.8235	4	3	1.4378	0.5783	0.2941	0.3137	0.6341	0.1442	0.3045	0.2809	0.3940
BrcSSR44	0.7222	5	4	1.7753	0.8136	0.3333	0.4492	0.6184	0.1543	0.4367	0.3930	0.0420
BrcSSR45	0.6250	4	4	2.1603	0.9705	0.7500	0.5544	0.4745	0.2769	0.5371	0.4794	0.3320
BrcSSR46	0.8438	2	2	1.3581	0.4334	0.3125	0.2722	0.6679	0.1243	0.2637	0.2289	1.0000
BrcSSR47	0.4667	5	4	2.6946	1.1056	0.3333	0.6506	0.8129	0.0575	0.6289	0.5570	0.0010
BrcSSR48	0.6389	4	4	2.0903	0.9394	0.5556	0.5365	0.4675	0.2848	0.5216	0.4633	0.0510
BrcSSR49	0.9722	2	2	1.0571	0.1269	0.0556	0.0556	0.4857	0.2647	0.0540	0.0526	1.0000
BrcSSR50	0.8333	4	4	1.4179	0.6191	0.3333	0.3032	0.4346	0.3253	0.2948	0.2797	1.0000
BrcSSR51	0.7188	4	3	1.7840	0.7731	0.3125	0.4536	0.7507	0.0830	0.4395	0.3934	0.1350
BrcSSR52	0.6765	5	4	1.9727	0.9231	0.4118	0.5080	0.6451	0.1376	0.4931	0.4473	0.3640
BrcSSR53	0.5385	2	2	1.9882	0.6902	0.9231	0.5169	0.5481	0.2061	0.4970	0.3735	0.0060
BrcSSR54	0.9722	2	2	1.0571	0.1269	0.0556	0.0556	0.4857	0.2647	0.0540	0.0526	1.0000
BrcSSR55	0.8889	2	2	1.2462	0.3488	0.2222	0.2032	0.4375	0.3214	0.1975	0.1780	1.0000
BrcSSR56	0.5625	3	3	2.2555	0.9126	0.8750	0.5746	0.4014	0.3728	0.5566	0.4742	0.0110
BrcSSR57	0.8889	3	3	1.2558	0.4258	0.1111	0.2095	0.7273	0.0937	0.2037	0.1939	0.0580
BrcSSR58	0.6154	3	2	1.8989	0.6663	0.4615	0.4923	0.7702	0.0746	0.4734	0.3613	1.0000
BrcSSR59	0.5625	2	2	1.9692	0.6853	0.8750	0.5081	0.3505	0.4632	0.4922	0.3711	0.0080
BrcSSR60	0.9118	2	2	1.1918	0.2984	0.1765	0.1658	0.6687	0.1239	0.1609	0.1480	1.0000
Mean	0.7524	3	2.7	1.6387	0.5885	0.4136	0.3571	0.5581	0.1980	0.3456	0.2970	0.5706

Note: The above shown for each SSR were the major allele frequency (MAF), genotype number (Gn), number of alleles detected (Na), effective number of alleles (Ne), Shannon’s Information index (I), observed heterozygosity (Ho), expected heterozygosity (He), F-Statistics (Fst), Gene flow estimated from Fst = 0.25(1 - Fst)/Fst (Nm), gene diversity (Gd), polymorphism information content (PIC) and Chi-square test for Hardy-Weinberg equilibrium (P_HW_).

### The identification and characteristic of SNPs in 10 NHCC accessions

A comparison of 10 NHCC accessions of five varieties with the Chinese cabbage genome was used to develop SNPs. To increase accuracy and minimize false-positive SNPs, we eliminated SNP sites that had missing data in any one of the 10 NHCC accessions. Finally, 1,228,979 SNP loci were identified, and the average SNP density in the whole genome was 4.33/Kb. This was greater than in tomato (0.6/Kb) and rice (1.7/Kb), but lower than in citrus (6.1/Kb) and potato (11.5/Kb) [31]. All SNPs identified in this study have been submitted to the nhccdata website (http://nhccdata.njau.edu.cn/).

**Figure 4:**
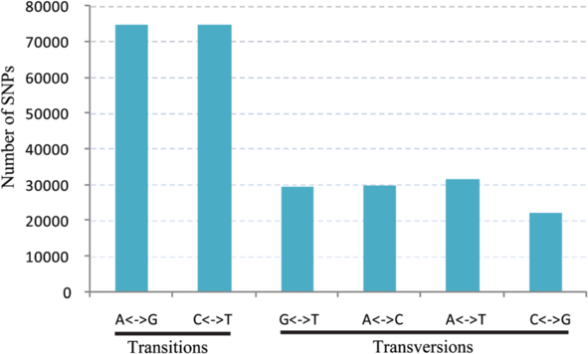
The average transitions and transversions of 10 non-heading Chinese cabbage accessions occurring within a set of 262,885 SNPs.

The number of SNPs for each accession ranged from 341,939 to 591,586. The average heterozygous ratio of the SNPs was ~42.53%, and the heterozygous ratio ranged from 18.92% to 65.07% among 10 NHCC accessions. An average of 189,666 SNPs was identified in coding domain sequences. The number of non-synonymous SNPs ranged from 47,178 to 85,510, with an average of 66,965 (Table 4). Of the identified SNPs, excluding those that were heterozygous, an average ~56.88% of SNPs belonged to the transition type in the 10 NHCC. The transition/transversion ratio can be used to measure the genetic distances. Generally, the higher transition/transversion ratio, the lower genetic divergence between two species. The high ratio of 1.32 between the NHCC and Chinese cabbage revealed the relatively low level of polymorphisms between them. A relatively high frequency of C/T alleles was identified, which was also observed in citrus, eggplant, and bean (Figure 4, Additional file 1: Table S11) [31-33].

**Table 4:** **Summary of SNPs in genomic and gene sequences of 10 non-heading Chinese cabbage accessions**

**Accession**	**SNP number**	**Het SNPs**	**Het ratio**	**Cds-SNP**	**Non-syn SNP**
NHCC001	591,586	242,686	41.02	242,590	85,346
NHCC026	406,117	76,831	18.92	171,343	59,793
NHCC006	505,378	240,376	47.56	195,893	69,939
NHCC007	519,509	219,735	42.30	200,549	71,400
NHCC008	497,659	244,327	49.10	190,314	67,839
NHCC013	348,319	96,220	27.62	138,324	48,357
NHCC009	591,404	384,799	65.07	243,708	85,510
NHCC015	341,939	140,946	41.22	134,752	47,178
NHCC010	551,325	285,881	51.85	218,500	77,623
NHCC029	349,403	141,990	40.64	160,691	56,663
Average	470,263.90	207,379.10	42.53	189,666.40	66,964.80

### The excavation of unique SNPs and genes from five NHCC varieties

The five varieties of NHCC have their own morphological characteristics. The variety-related SNPs and genes were quickly and accurately identified using the varieties genomic information. Based on the genotypes and phenotypes of the five varieties, the genes associated with variety-related traits were uncovered. For example, by comparing the Tillering cabbage and other four varieties, genes associated with tillering were identified. Similarly, the flowering and early bolting genes were identified by comparing the flowering Chinese cabbage variety and other varieties. Additionally, we have detected the expression of variety-specific genes at the transcriptome level. The functional annotation and the metabolic networks were also conducted for differentially expressed genes (DEGs).

At the genomic level, we identified variety-specific SNPs. The non-synonymous SNPs could directly change the encoded amino acid, which could change the function of the protein. Therefore, we surveyed the non-synonymous SNPs in each accession. To better analyze the point mutations, which ranged from 1,133 to 2,104 in the five varieties, we exploited the variety-specific non-synonymous SNPs. These SNPs were located in 710 to 1,107 genes of the five varieties. Transcriptome data were used to identify 897, 651, 970, 1,247, and 699 genes in NHCC001, NHCC006, NHCC008, NHCC009, and NHCC010, respectively (Additional file 1: Table S12). Then, the variety-specific DEGs were identified, whose expression levels were 0.5 or 2 times expression level than each of other varieties. A total of 189 variety-specific DEGs were discovered, consisting of 28, 1, 45, 26, and 2 low expressing genes and 34, 5, 24, 11, and 13 high expressing genes in the five varieties, respectively.

**Figure 5:**
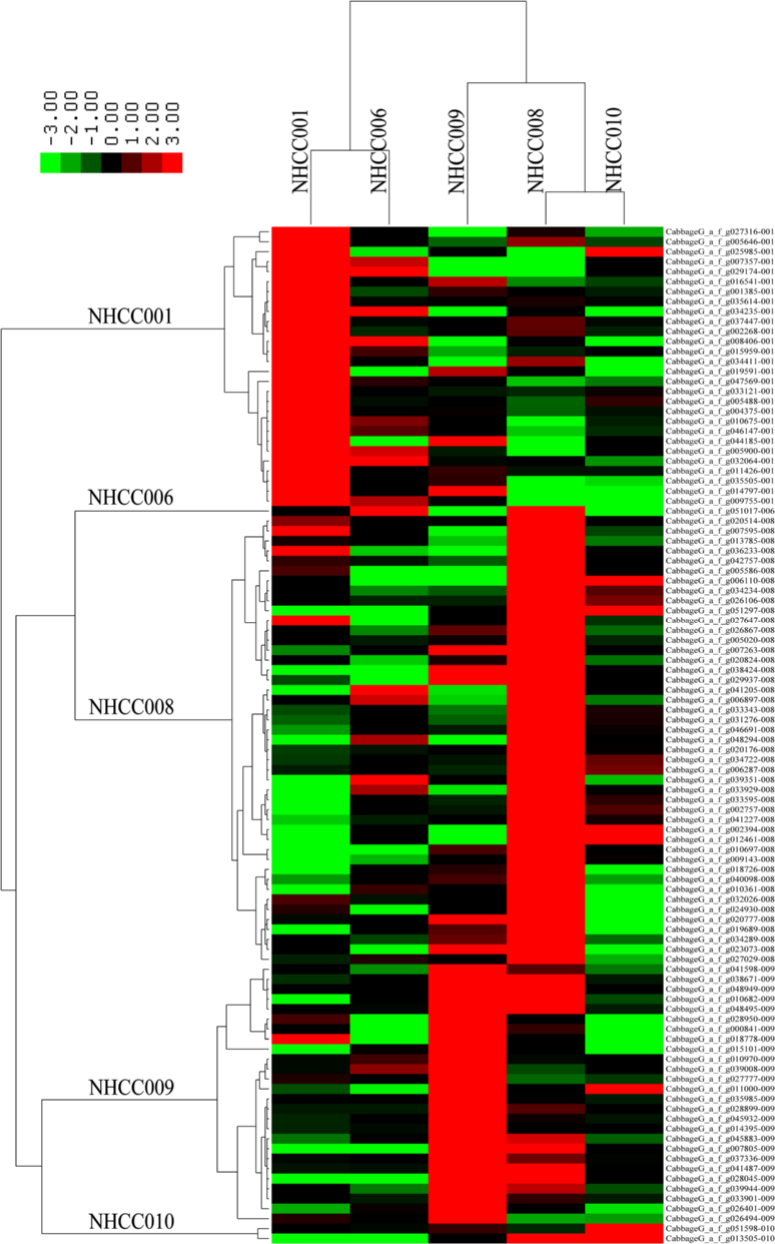
Expression profile of variety-specific, high-level, differentially expressed non-heading Chinese cabbage genes. The expression levels of all genes identified in this study were measured by transcriptome data from the five NHCC varieties. Hierarchical clustering was used to represent the gene expression for each variety.

**Figure 6:**
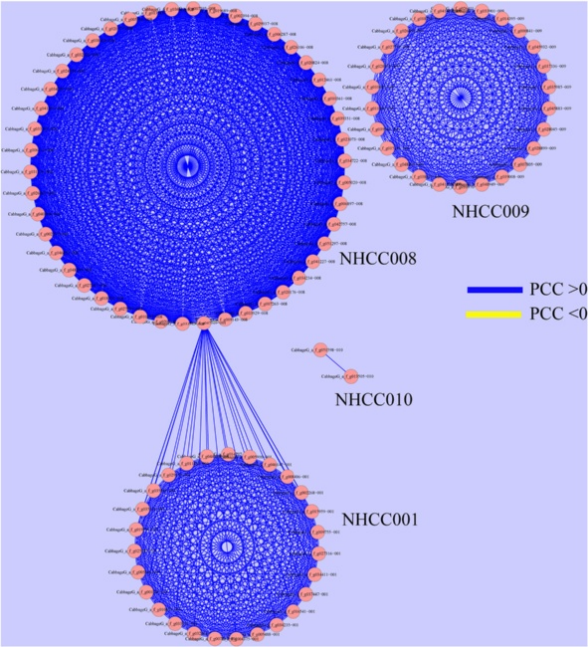
The interaction network of variety-specific, high-level, differentially expressed non-heading Chinese cabbage genes. The Pearson’s correlation coefficients were calculated according to the transcriptome data of the five NHCC varieties.

To obtain a more intuitive understanding of the relationship among these DEGs, clustering analyses were carried out based on the expression level. The high expressing DEGs could be divided into five groups, corresponding to the five varieties (Figure 5), while low expressing DEGs did not completely cluster based on variety (Additional file 5: Figure S4). Furthermore, the relationships among these genes was studied using Cytoscape software. Finally, the absolute Pearson’s correlation coefficients of the 1,662 gene pairs were greater than 0.8 in the high expressing DEGs. Most genes had positive relationships, except the *CabbageG_a_f_g047569*, *CabbageG_a_f_g033595*, and *CabbageG_a_f_g009143* genes. These genes could be divided into four groups, corresponding to the four varieties. Only one gene was identified in NHCC006, so it was not involved in the network (Figure 6). The relationships among low expressing genes were complex, with 221 negative- and 373 positive-related gene pairs (Additional file 6: Figure S5). In addition, 673 negative- and 3,377 positive-related gene pairs existed in the high and low expressing genes, respectively (Additional file 7: Figure S6).

Using strict standards, which defined the expression level of the gene as 0.2 or 5 times the lowest or highest expression, respectively, of the other varieties, 33 variety-specific DEGs were identified. Of which, 15, 9, 8, and 1 genes were found in NHCC001, NHCC009, NHCC008, and NHCC010, respectively, while none was identified in NHCC006. The analysis of Pearson’s correlation coefficients showed that 15 negative- and 94 positive-related gene pairs were present in these genes, and the *CabbageG_a_f_g013270* gene existed in more negative gene pairs than any other genes (Additional file 8: Figure S7).

**Figure 7:**
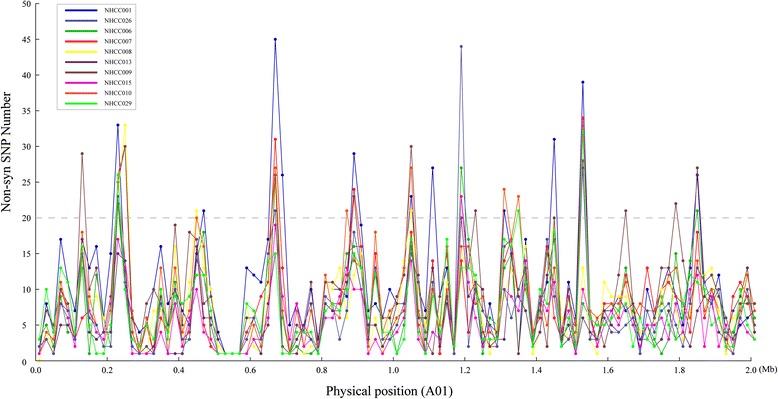
The density of non-synonymous SNPs in 10 non-heading Chinese cabbage accessions on chromosomes. The number of non-synonymous SNPs was calculated for each dot using 20-Kb windows. The figure shows a fragment of chromosome 1(A01: 0–2 Mb) as an example.

For a more intuitive presentation of these non-synonymous SNPs, we plotted their distribution on the chromosomes (Figure 7), revealing that their distributions were different in each accession. This may be because of differential selection during the breeding process. In general, regions with more non-synonymous mutations were often the subject of selection. In 10 NHCC accessions, 3,228 regions with a total length of 20 Kb were identified. The number of non-synonymous SNPs was greater than 20 in these regions. The number of these regions was different on each chromosome, ranging from 21 (A10) to 720 (A03). In addition, we mapped the density of non-synonymous SNPs on the chromosomes for each accession (Additional file 9: Figure S8).

### The evolutionary relationship of 10 NHCC accessions by SNP markers

**Figure 8:**
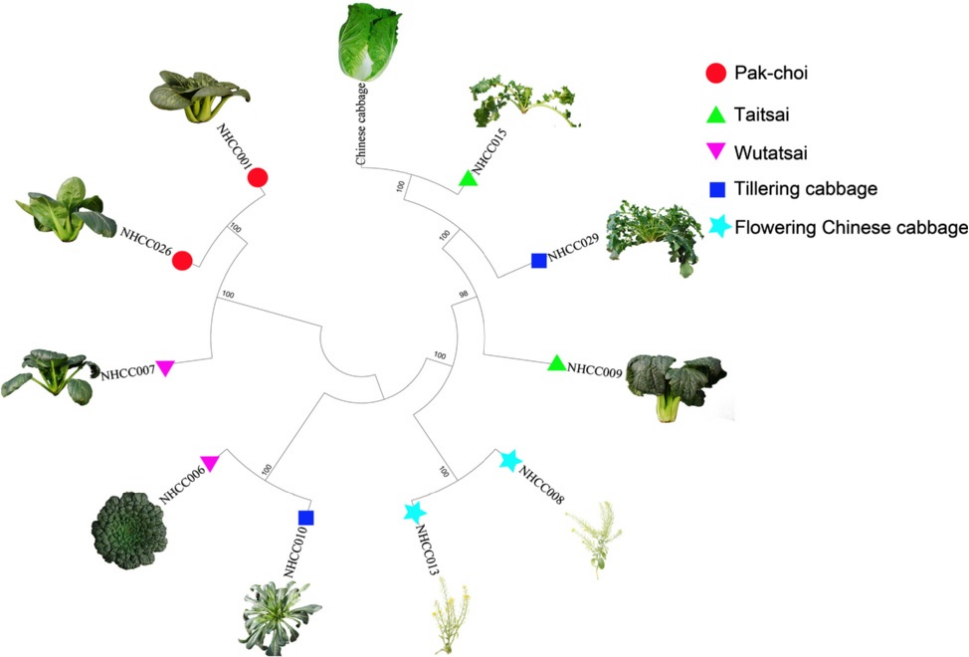
Neighbor-joining phylogenetic tree of 10 non-heading Chinese cabbage accessions (five varieties) and Chinese cabbage constructed by MEGA5 using SNPs in the coding domain sequences. The numbers are bootstrap values based on 1,000 iterations. Only bootstrap values larger than 50 are indicated.

To understand the phylogenetic relationships causing morphological diversity in NHCC, a neighbor-joining phylogenetic tree was constructed by MEGA5 using 10 NHCC accessions and Chinese cabbage Chiifu-401-42 [34]. The SNPs located in the coding domain sequences, excluding the missing site, were used to construct the phylogenic tree (Figure 8). In the phylogenetic tree, two accessions of Pak-choi, NHCC001 and NHCC026, and flowering Chinese cabbage, NHCC008 and NHCC013, clustered together. The Taitsai (NHCC015) had a close relationship with Chinese cabbage. Although NHCC010 and NHCC029 belonged to the Tillering cabbage, they did not cluster together. The previous classification might be only based on the tiller, which affected by only a few genes. Thus, they did not cluster together in this tree whose construction was based on genome-wide SNPs. The NHCC010 and NHCC006, which share land collapse and short plant height characteristics, clustered together. Additionally, NHCC029, which shares similar traits with NHCC015, clustered together. These phenomena indicated that the morphological classification might be based on one or several distinct external plant characteristics. However, classification should be determined by the internal genes, coupled with complex environmental interactions. Therefore, the traditional morphological classification might be erroneous. Currently, we can correct traditional morphological classifications through whole-genome sequencing and re-sequencing, furthering the understanding of the NHCC.

## Discussion

### Efficient and strict flow chart for identification of SSR and SNP markers

In this study, our major aims were to find a large set of accurate SSR and SNP markers in the NHCC, and to gain further insight into the genetic diversity and relationships among representative cultivars and related species. We analyzed the distribution and frequency of microsatellites with mono- to nonanucleotide motifs. To find more accurate SSRs, we used the strict standard that the total SSR length is not less than 18 bp. Thus, the results of this study may differ from previous studies. When compared with previous research, the results obtained could differ because of the following aspects: (1) different search parameters, including the different minimum length (no less than 18 bp versus 12 bp), and different repeat types (mono- to nonanucleotide versus di- to octanucleotide or another range); (2) different software and algorithms used for the SSR search (MISA versus SSRtool); (3) the data used for SSR detection was of a different size and version; and (4) the different analytical methods and manifestations used (count/Mb versus length/Mb). These seemingly minor differences in procedure could strongly influence microsatellite distributions and comparisons among studies. For the development of SNP markers, errors in sequencing or assembling of the NHCC genome also might lead to false SNPs. Therefore, it is important to consider the above-mentioned points when we compared the SSR or SNP frequency and density generated by different genome datasets or research groups.

### Genetic relationship analysis of 18 *Cruciferae* species

The 14 NHCC accessions and four other *Cruciferae* species were analyzed using SSR markers. The analyses of a dendrogram and population structure, as well as PCA, revealed four clusters. Although the research did not completely distinguish the five NHCC varieties, which may have been because of the limited number of SSR markers used for the genetic analyses, it accurately separated NHCC, Chinese cabbage, broccoli, and *Arabidopsis*. Thus, a larger number of SNP markers were used to construct the phylogenic tree. Both of the SSR and SNP marker analyses revealed that the Taitsai variety (NHCC015 and NHCC009) had a close relationship with Chinese cabbage. It was also consistent with the theory that Chinese cabbage was derived from a hybrid of Taitsai and turnip [35]. The Pak-choi, flowering Chinese cabbage, and Taitsai varieties could be distinguished using the SNP markers. The classification of Tillering cabbage and Wutatsai might be only based on one or several distinct phenotypic plant characteristics; thus, we attempted to distinguish them using whole genome SNPs. Classification only based on morphology may be problematic, and a true classification should be determined using the internal genes of the whole genome, coupled with the complex environmental factors. Currently, it is possible for us to adjust traditional morphological classifications using the SSRs and SNPs of the whole genome. Furthermore, these markers developed in our study can be useful for population structure analyses of NHCC and other related species in the future.

### Use of new SSR and SNP markers for NHCC and *Cruciferous* species research

It was important to develop molecular markers to investigate genetic variability and explore genome evolutionary. Until now, only a few low-density genetic maps have been constructed owing to lack of highly polymorphic and reliable molecular markers in NHCC. In addition, most linkage maps with important agronomic trait loci have been developed with primarily low-throughput markers, such as AFLP, RFLP, and RAPD or a few SSR markers. The development of these markers is time consuming, labor intensive, and expensive. Thus, only a few economically important genes had been identified using a map-based cloning strategy in NHCC [36], suggesting that marker-assisted selection breeding was still not well developed compared with in other horticultural species, such as cucumber [37]. SSR or SNP markers have proven to be useful markers in the population genetic studies of species [25,38,39]. Currently, with the development of bioinformatics and the next-generation sequencing technology, it is very convenient and feasible to obtain a large number of SSR and SNP markers by genome sequencing. In this study, we developed a large number of SSR and SNP markers, and obtained their exact physical positions in the NHCC genome. We designed primer pairs for NHCC SSRs, and verified the polymorphism by polymerase chain reaction (PCR) and gel electrophoresis in some important *Cruciferous* species. NHCC had a relatively large level of morphological and genetic polymorphisms, and SNPs were identified in different varieties. In our study, the SNPs were classified according to the five varieties. Variety-specific genes were also identified and verified using the transcriptome. These genes might be useful for distinguishing the five varieties of NHCC.

## Conclusions

NHCC is an ecologically important vegetable crop in Southeast Asia, Japan, USA, and Europe. However, the insufficient genomic and transcriptome data in public databases have limited our understanding of the molecular mechanisms underlying the adaptation of NHCC. With the development of high-throughput genome sequencing technology, it is now possible to uncover large numbers of DNA markers. This work contributed to a detailed characterization of 20,836 SSRs and 1,228,979 SNPs in NHCC and compared them with markers in other representative species. For the SSR markers, dinucleotide repeats were the most frequent SSRs in the genome. While the frequency of trinucleotide repeats were much higher than dinucleotides in gene sequences. Primers for the SSRs in the gene sequences of NHCC were designed, and the SSR polymorphisms were verified using PCR. The results showed that the SSR markers were highly polymorphic among the 18 *Cruciferous* species. By comparing NHCC with Chinese cabbage, a large number of SNP markers were identified in the five NHCC varieties. The potential variety-specific related genes identified lay a solid foundation for further investigations into comparative genome analyses among the five varieties. Furthermore, they will be useful for further functional genomic studies in the *Brassica* genus. These SNP and SSR markers will be valuable genomic resources for future *Cruciferous* research and breeding applications.

## Methods

### Plant materials and DNA preparation

Genomic DNA for SNP and SSR analysis was extracted from leaves of 5-week-old seedlings using a Plant DNA extraction kit (Qiagen, Beijing, China). Ten NHCC accessions were used for the development of SNP markers, and the validation of polymorphic SSR markers used 18 accessions, which were from 14 NHCC, 1 Chinese cabbage, 2 broccoli, and 1 *Arabidopsis*.

### The identification and characterization of SSR and SNP markers

The Microsatellite identification software MISA was used to identify SSRs in NHCC genome sequences (MISA, http://pgrc.ipk-gatersleben.de/misa/). The parameters were set as follows: monomers (×18), 2-mers (×9), 3-mers (×6), 4-mers (×5), 5-mers (×4), 6-mers (×4), 7-mers (×3), 8-mers (×3), and 9-mers (×3). This tool allowed for the identification and localization of perfect microsatellites as well as compound microsatellites. The maximum size of interruption allowed between two different SSRs in a compound sequence is 100 bp.

The identification of SNPs between the 10 accessions and the reference genome (Chiifu-401-42) was performed using the SOAPsnp software as previously reported [40]. In SNP calling, the quality threshold was set to 20, which corresponded to an error rate of less than 1%. The variety-specific SNPs were identified using a perl script. The distribution of different types of SSRs and SNPs on chromosomes was plotted using the SVG program written by a perl script. All SNP and SSR markers identified in this study have been submitted to the nhccdata website (http://nhccdata.njau.edu.cn/).

### Primer design for SSR markers

The SSRs of the gene sequences were used for primer design by Primer3 program [41]. The parameters of Primer3 were set as follows: (a) Primer length from 18 to 27 bases, with an optimum size of 20 nt. (b) The melting temperature (Tm) ranged from 55°C to 65°C with an optimum temperature of 60°C. (c) The predicted target PCR products ranged from 100 to 280 bp, with an optimum product size of 150 bp, and all other parameters were set to the default values. Subsequently, the results from Primer3 were further filtered to minimize the chance of encompassing tandem repeats, and self- or pair complementation in the experiment.

### The assessment of SSR polymorphisms

A total of 74 primers were selected from the newly designed primers of the gene SSR markers and used to detect SSR polymorphisms among the 18 species. Their sequences were listed in Additional file 1. The selected primers were synthesized by Invitrogen Biotech (Shanghai, China). All PCRs were conducted in 20-μL reaction mixtures containing 50 ng of genomic DNA, 0.5 U of Taq DNA polymerase (TaKaRa, Dalian, China), 0.4 μM primer, 1× PCR Buffer, 25 μM of dNTPs, and 1.5 mM MgCl_2_. SSR loci were amplified using Thermal Cycler (Eppendorf, Shanghai, China), and the following program was used: 5 min initial denaturation at 95°C; 35 cycles of 30s at 95°C, 30s at the appropriate annealing temperature, 45 s of extension at 72°C, and 10 min at 72°C for final elongation. Finally, the PCR products were initially assessed for size polymorphisms on 6% denaturing polyacrylamide gels and then visualized by silver nitrate staining.

The genotyping data were subsequently used to determine genetic relationships among the 18 accessions assessed. The genetic distance were calculated according to Nei’s unbiased measures using the POPGEN1.32 software (http://www.ualberta.ca/~fyeh/popgene_download.html). The PCA and the dendrogram construction were performed based on the unweighted pair-group method with arithmetic average using the NTSYS software [42]. The confidence of branch support was then evaluated by a bootstrap analysis with 1,000 replicates using Free Tree [43].

The number of alleles, observed heterozygosity, expected heterozygosity, gene flow, and Shannon’s Information index were calculated using POPGEN1.32. The major allele frequency, chi-square test for Hardy–Weinberg equilibrium allele frequencies, genetic diversity, and PIC were calculated using PowerMarker3.25 [44]. The Structure2.3.4 software was used to investigate the population structure with the number of populations ranging from 2 to 9. Both the length of the burn-in period and the number of the Markov Chain Monte Carlo reps after burn-in were set to 100,000 [45].

### DEGs identified by RNA-sequencing

The transcriptome data of five varieties was obtained from our laboratory (http://nhccdata.njau.edu.cn/). Leaf tissues of *B. rapa* accessions were collected from 7-week-old plants, which were grown under greenhouse conditions at 25°C. mRNA was prepared, and an individual cDNA library with insert sizes of 200 bp was constructed for each sample. The libraries were sequenced for paired-end reads of 90 bp on the Illumina Hiseq 2000 platform. FastQC was used to check and visualize the quality of the RNA-seq reads (http://www.bioinformatics.babraham.ac.uk/projects/fastqc/). The NGS QC Toolkit was used to remove the pair-end reads containing Ns or those where the number of bases whose PHRED-like score was less than 20 exceeded 10%. If the first 9 bp of filtered reads showed unstable base composition based on the percentages of the four different nucleotides, then they were trimmed before read mapping using TopHat. The uniquely mapped reads were used for subsequent analyses. The transcripts were constructed and the expression as fragments per kilobase of transcript sequence per millions base pairs (FPKM) values of transcripts were quantified in each sample using Cufflinks. The significance of DEGs was calculated using the software of IDEG6 [46], and a p-value of less than 0.01 was a DEG criterion. In this study, to avoid the potential noise signal from high-throughput sequencing, an absolute fold change of no less than 2.0 was used to define DEGs, including up-regulated and down-regulated genes. Furthermore, the expression patterns of DEGs were displayed using the heat-map function in the Cluster program, and the results were exhibited using Tree View [47].

### The annotation, biological process, pathway, and network analyses

The annotation of DEGs in NHCC was obtained by searching the protein databases Iprscan (http://www.ebi.ac.uk/Tools/pfa/iprscan/), UniProtKB (http://www.ebi.ac.uk/uniprot/), TrEMBL (http://www.ebi.ac.uk/uniprot/TrEMBLstats/), GO (http://www.geneontology.org/), and KEGG (http://www.genome.jp/kegg/). The annotations obtained from these five protein databases was integrated using perl script. In addition, the biological process and functions of the DEGs were also analyzed using the Gene Ontology database. The metabolomics, biological interpretation, and functional pathways of these DEGs were constructed by KEGG and STRING [48]. The interaction network of the DEGs was constructed using Cytoscape software according to the level of the genes [49]. The flower and glucosinolate genes of Chinese cabbage were downloaded from the *Brassica* database (http://brassicadb.org/) [2]. The distribution of flower, glucosinolate genes, and related SSR markers on chromosomes were plotted using a perl script.

## Availability of supporting data

All the supporting datasets have been submitted to the NHCC Data Center (http://nhccdata.njau.edu.cn/), including SNP (All_SNPs.data.tgz), SSR (All_SSRs.data.tgz), and transcriptome (Five_transcriptome.data.tgz) datasets.

## Additional files

Additional file 1: Table S1. Distribution of SSR repeats in genomic sequences of non-heading Chinese cabbage (NHCC) and selected plant species. **Table S2.** Distribution of SSR repeats in genomic sequences of non-heading Chinese cabbage (NHCC) and selected plant species (considering sequence complementary). **Table S3.** Frequency of mono- to nonanucleotide SSR repeat motifs in non-heading Chinese cabbage (NHCC) genome. **Table S4.** Summary of the SSRs distribution on chromosomes in the non-heading Chinese cabbage (NHCC) genome. **Table S5.** Distribution of mono- to nonanucleotide repeats in genomic sequences of non-heading Chinese cabbage (NHCC). **Table S6.** Distribution of mono- to nonanucleotide repeats in gene sequences of non-heading Chinese cabbage (NHCC). **Table S7** The summary of flower, glucosinolate genes, and related SSR markers on chromosomes in the non-heading Chinese cabbage (NHCC) genome. **Table S8.** Informativeness of 18 Cruciferae accessions used for assessing SSR polymorphisms. **Table S9.** Characteristics of 60 polymorphic SSR markers developed in non-heading Chinese cabbage (NHCC) (Chr =chromosome, Ta = annealing temperature, Size = size of cloned allele). **Table S10.** Nei’s unbiased measurements of genetic identity and genetic distance among 18 Cruciferae accessions. **Table S11.** Summary of transitions and transversions for SNPs between non-heading Chinese cabbage (NHCC) accession and Chinese cabbage (reference genome). **Table S12.** The summary of variety-specific genes and SNPs in the five varieties of non-heading Chinese cabbage (NHCC). Additional file 2: Figure S1. Distribution of SSR repeats in genomic sequences of non-heading Chinese cabbage and other selected plant species. Frequency values are expressed as number of repeats per million base pairs of sequence. Additional file 3: Figure S2. Histogram presentation of Gene Ontology classifications in non-heading Chinese cabbage. The results were summarized in three main categories: biological process, cellular component, and molecular function. The right y-axis indicates the number of genes in a category. The left y-axis indicates the number of unique sequences in a specific category. Additional file 4: Figure S3. Distribution of mono- to pentanucleotide repeats in the genomic sequences of non-heading Chinese cabbage and other selected plant species. (a) Distribution of mono- to trinucleotide repeats in genomic sequences of NHCC and other selected plant species. Frequency values are expressed as number of repeats per million base pairs of sequence. (b) Distribution of tetranucleotide repeats in genomic sequences of NHCC and other selected plant species. Frequency values are expressed as number of repeats per million base pairs of sequence. (c) Distribution of pentanucleotide repeats in genomic sequences of NHCC and other selected plant species. Frequency values are expressed as number of repeats per million base pairs of sequence. Additional file 5: Figure S4. Expression profile of variety-specific, low-level, differentially expressed non-heading Chinese cabbage. The expression levels of the genes identified in this study were measured by transcriptome data in the five NHCC varieties. Hierarchical clustering is used to represent the gene expression levels for each variety. Additional file 6: Figure S5. The interaction network of variety-specific, low-level, differentially expressed, non-heading Chinese cabbage genes. The Pearson’s correlation coefficients were calculated according to the transcriptome data of the five NHCC varieties. Additional file 7: Figure S6. The interaction network of variety-specific, differentially expressed, non-heading Chinese cabbage genes. The Pearson’s correlation coefficients were calculated according to the transcriptome data of the five NHCC varieties. Additional file 8: Figure S7. The interaction network of variety-specific, differentially expressed, non-heading Chinese cabbage genes identified using a strict criterion. The Pearson’s correlation coefficients were calculated according to the transcriptome data of the five NHCC varieties. Additional file 9: Figure S8. The density of non-synonymous SNPs in non-heading Chinese cabbage accession on 10 chromosomes. The number of non-synonymous SNPs was calculated using 20 Kb windows.

## Abbreviations

NHCC: Non-heading Chinese cabbage
SSRs: Simple sequence repeats
SNPs: Single nucleotidepolymorphisms
QTL: Quantitative trait locus
RFLP: Restriction fragment length polymorphism
RAPD: Random amplified polymorphism DNA
AFLP: Amplified fragment length polymorphism
STSs: Sequence tag sites
PIC: Polymorphism information content
PCA: Principal component analysis
DEGs: Differentially expressed genes
PCR: Polymerase chain reaction
FPKM: Fragments per kilobase of transcript sequence per millions base pairs
