# Supplementary figures and images for: Genome-wide identification of SSR and SNP markers from the non-heading Chinese cabbage for comparative genomic analyses
Source: BMC Genomics. 2015 Apr 20;16(1):328. doi: 10.1186/s12864-015-1534-0 (PMC4408590; doi:10.1186/s12864-015-1534-0)

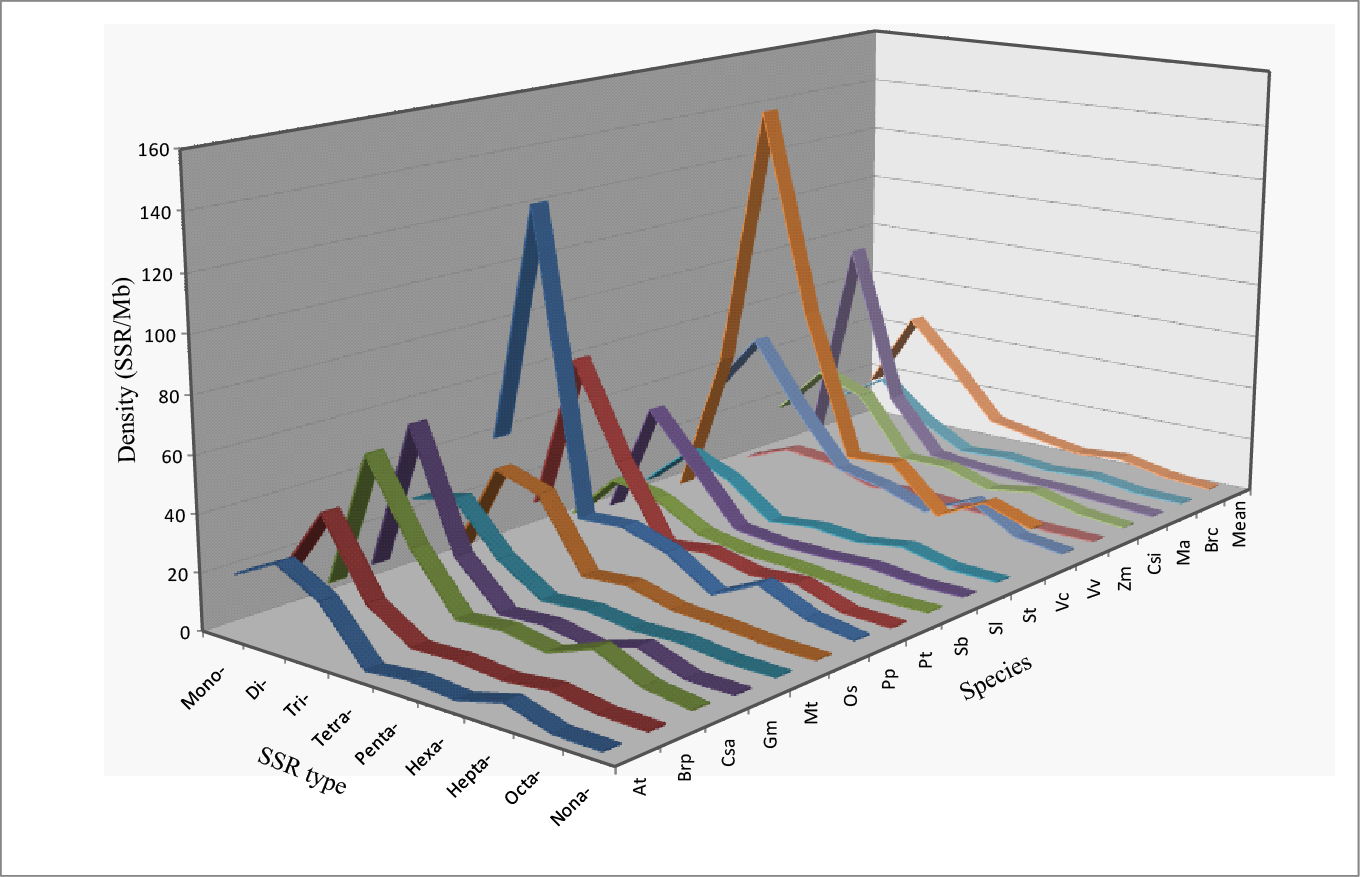

Supplement: Additional file 2: Figure S1. — Distribution of SSR repeats in genomic sequences of non-heading Chinese cabbage and other selected plant species. Frequency values are expressed as number of repeats per million base pairs of sequence. [file 12864_2015_1534_MOESM2_ESM.png]

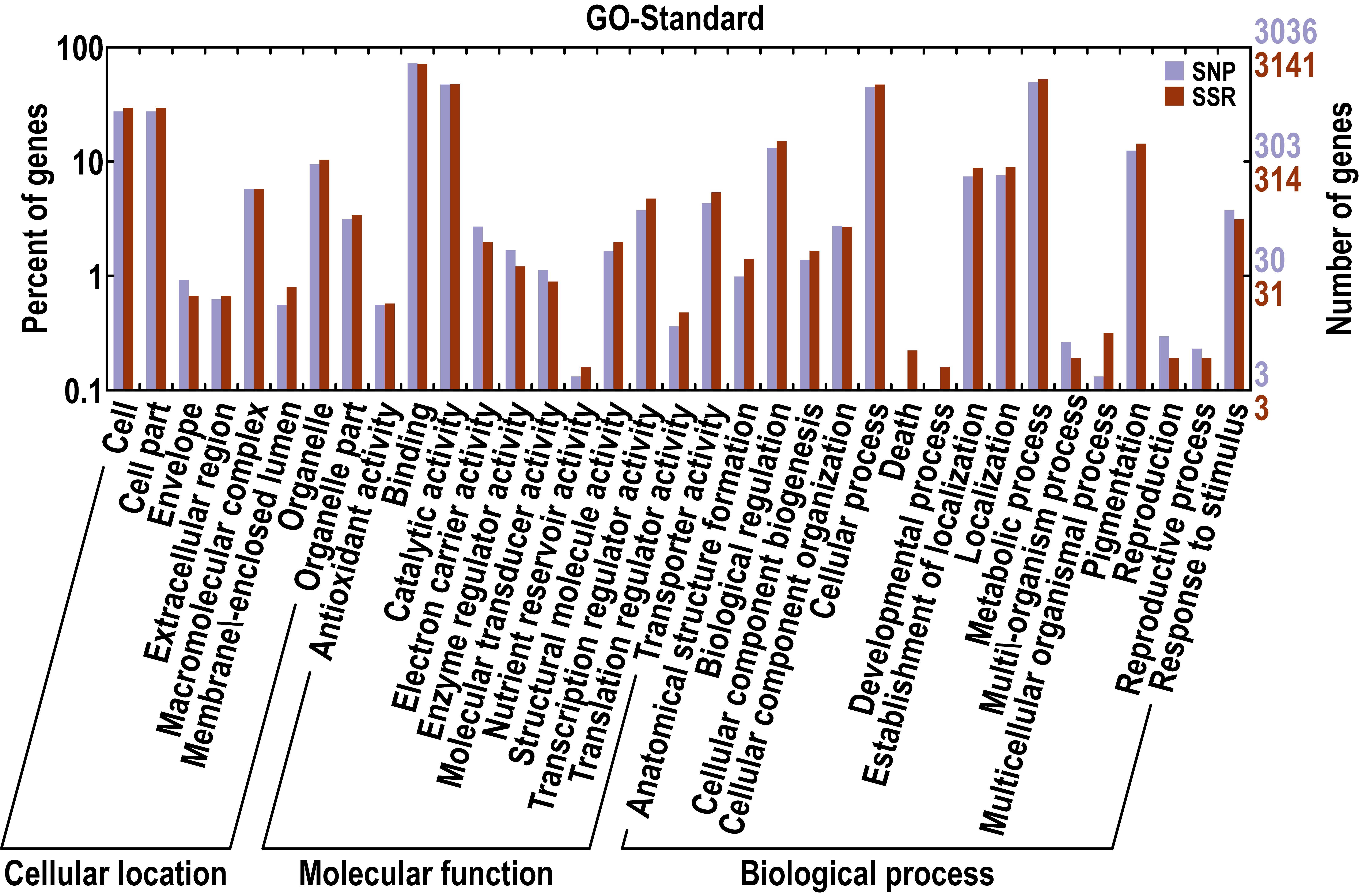

Supplement: Additional file 3: Figure S2. — Histogram presentation of Gene Ontology classifications in non-heading Chinese cabbage. The results were summarized in three main categories: biological process, cellular component, and molecular function. The right y-axis indicates the number of genes in a category. The left y-axis indicates the number of unique sequences in a specific category. [file 12864_2015_1534_MOESM3_ESM.png]

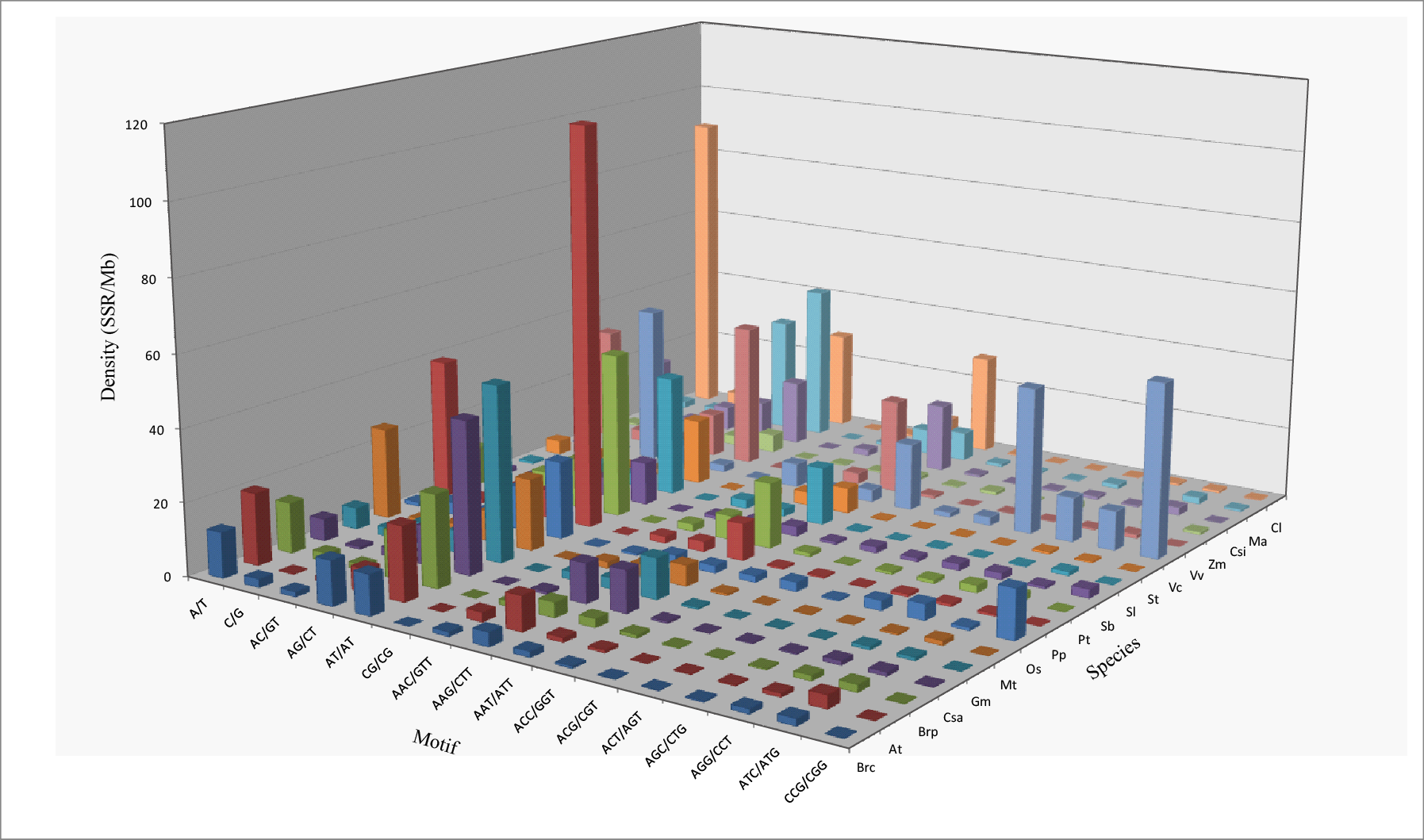

Supplement: Additional file 4: Figure S3. — Distribution of mono- to pentanucleotide repeats in the genomic sequences of non-heading Chinese cabbage and other selected plant species. (a) Distribution of mono- to trinucleotide repeats in genomic sequences of NHCC and other selected plant species. Frequency values are expressed as number of repeats per million base pairs of sequence. (b) Distribution of tetranucleotide repeats in genomic sequences of NHCC and other selected plant species. Frequency values are expressed as number of repeats per million base pairs of sequence. (c) Distribution of pentanucleotide repeats in genomic sequences of NHCC and other selected plant species. Frequency values are expressed as number of repeats per million base pairs of sequence. [file 12864_2015_1534_MOESM4_ESM.zip › add4/add4/Figure S3a.png]

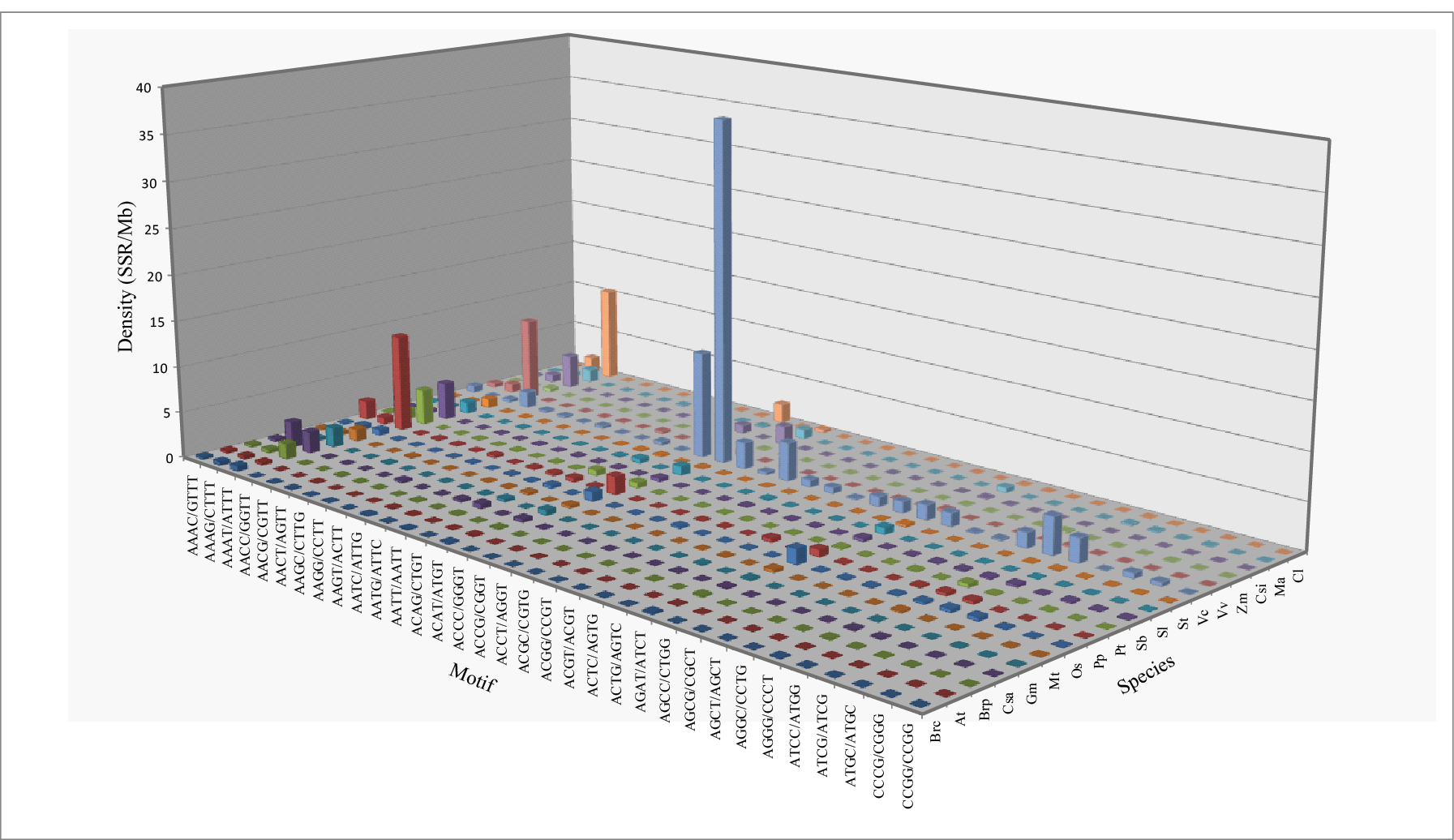

Supplement: Additional file 4: Figure S3. — Distribution of mono- to pentanucleotide repeats in the genomic sequences of non-heading Chinese cabbage and other selected plant species. (a) Distribution of mono- to trinucleotide repeats in genomic sequences of NHCC and other selected plant species. Frequency values are expressed as number of repeats per million base pairs of sequence. (b) Distribution of tetranucleotide repeats in genomic sequences of NHCC and other selected plant species. Frequency values are expressed as number of repeats per million base pairs of sequence. (c) Distribution of pentanucleotide repeats in genomic sequences of NHCC and other selected plant species. Frequency values are expressed as number of repeats per million base pairs of sequence. [file 12864_2015_1534_MOESM4_ESM.zip › add4/add4/Figure S3b.png]

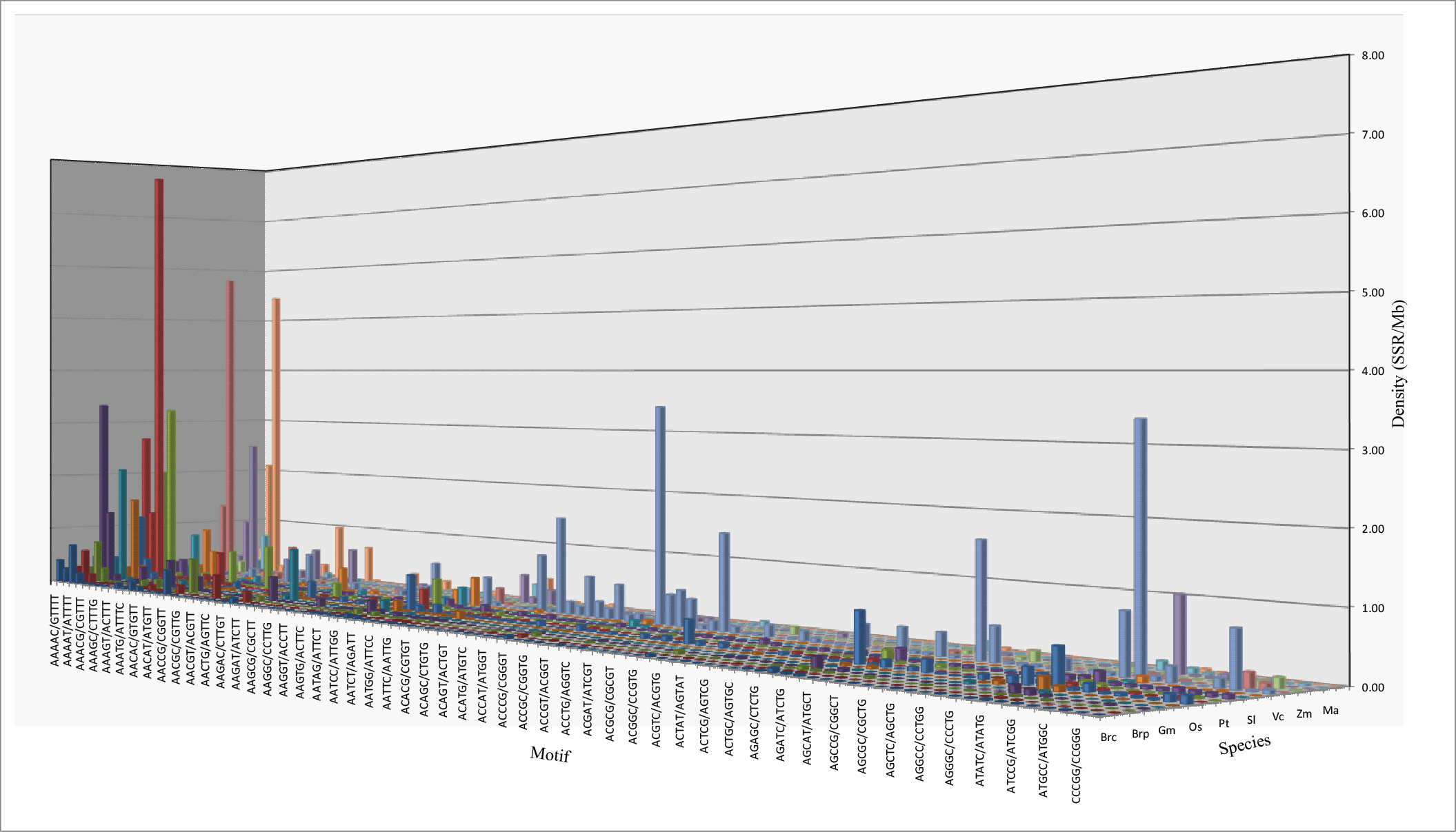

Supplement: Additional file 4: Figure S3. — Distribution of mono- to pentanucleotide repeats in the genomic sequences of non-heading Chinese cabbage and other selected plant species. (a) Distribution of mono- to trinucleotide repeats in genomic sequences of NHCC and other selected plant species. Frequency values are expressed as number of repeats per million base pairs of sequence. (b) Distribution of tetranucleotide repeats in genomic sequences of NHCC and other selected plant species. Frequency values are expressed as number of repeats per million base pairs of sequence. (c) Distribution of pentanucleotide repeats in genomic sequences of NHCC and other selected plant species. Frequency values are expressed as number of repeats per million base pairs of sequence. [file 12864_2015_1534_MOESM4_ESM.zip › add4/add4/Figure S3c.png]

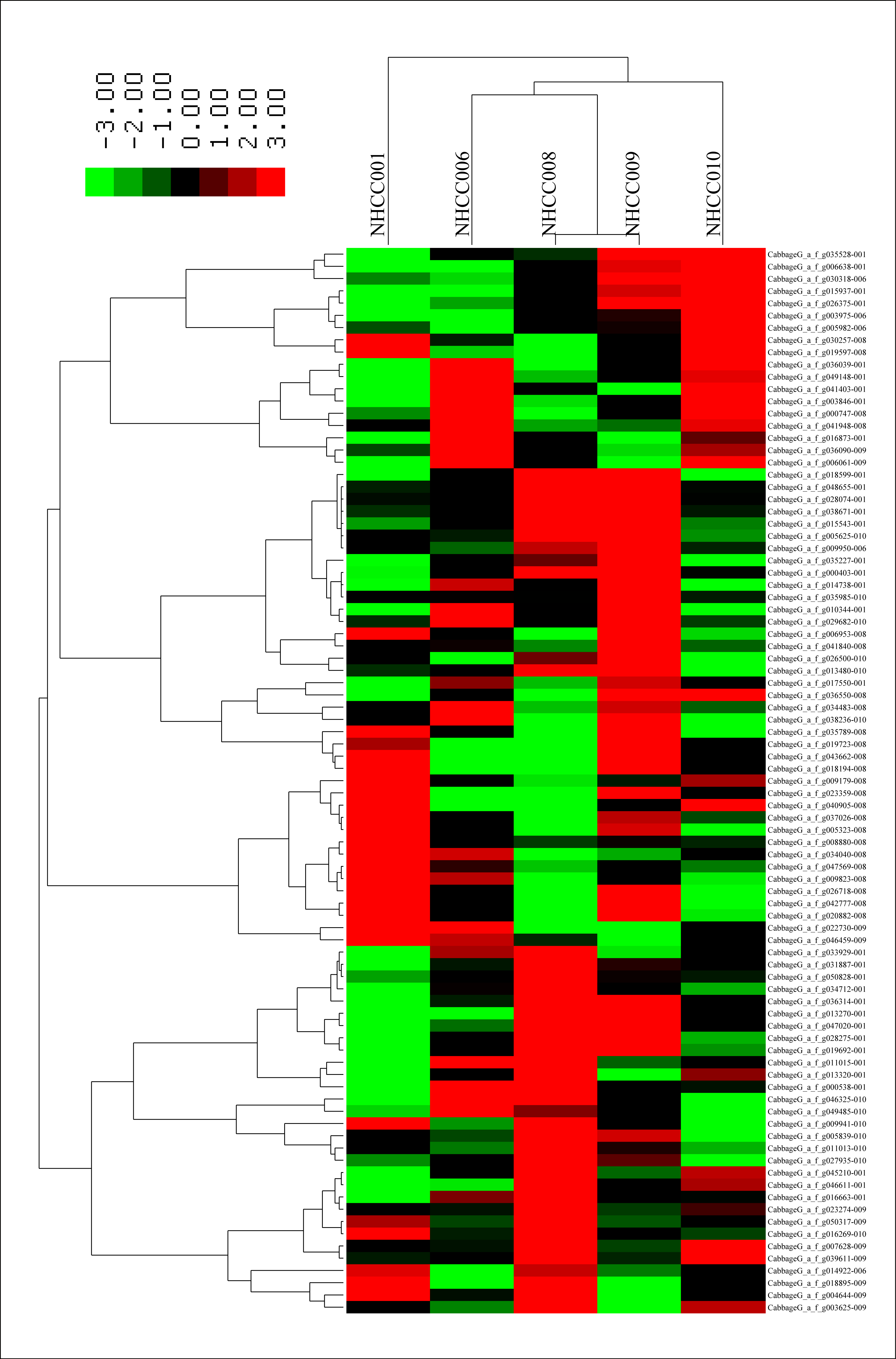

Supplement: Additional file 5: Figure S4. — Expression profile of variety-specific, low-level, differentially expressed non-heading Chinese cabbage. The expression levels of the genes identified in this study were measured by transcriptome data in the five NHCC varieties. Hierarchical clustering is used to represent the gene expression levels for each variety. [file 12864_2015_1534_MOESM5_ESM.png]

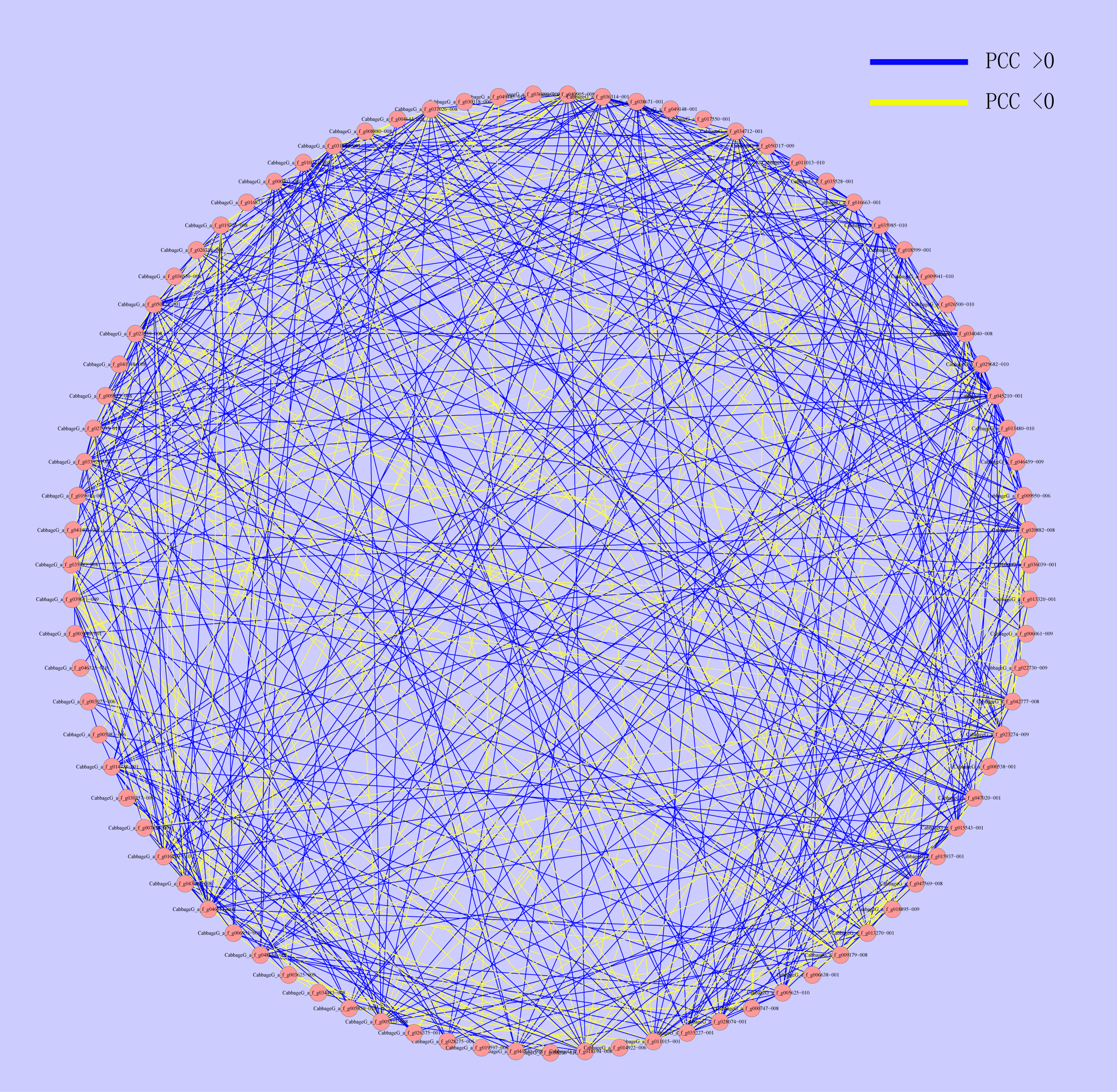

Supplement: Additional file 6: Figure S5. — The interaction network of variety-specific, low-level, differentially expressed, non-heading Chinese cabbage genes. The Pearson’s correlation coefficients were calculated according to the transcriptome data of the five NHCC varieties. [file 12864_2015_1534_MOESM6_ESM.png]

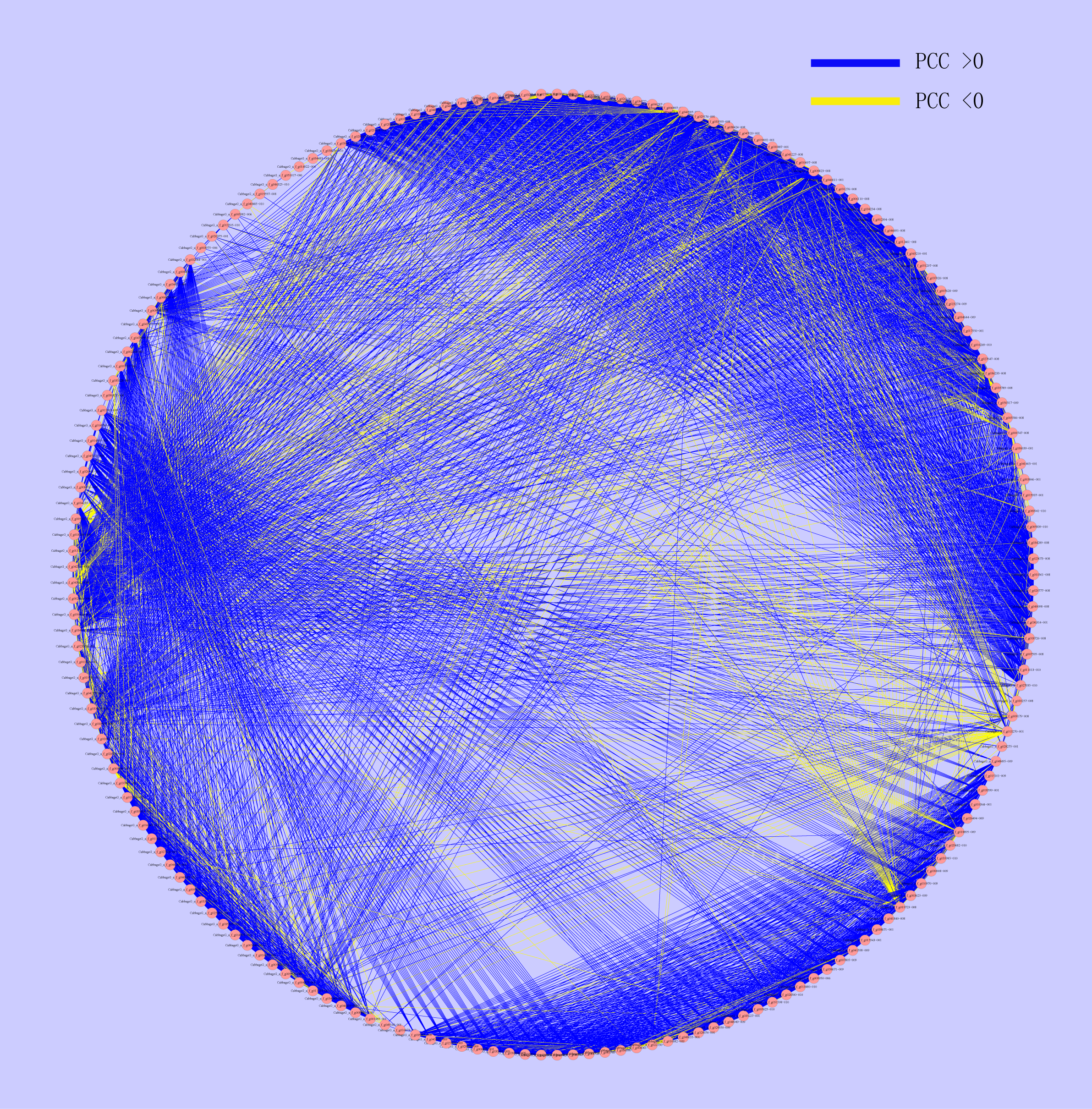

Supplement: Additional file 7: Figure S6. — The interaction network of variety-specific, differentially expressed, non-heading Chinese cabbage genes. The Pearson’s correlation coefficients were calculated according to the transcriptome data of the five NHCC varieties. [file 12864_2015_1534_MOESM7_ESM.png]

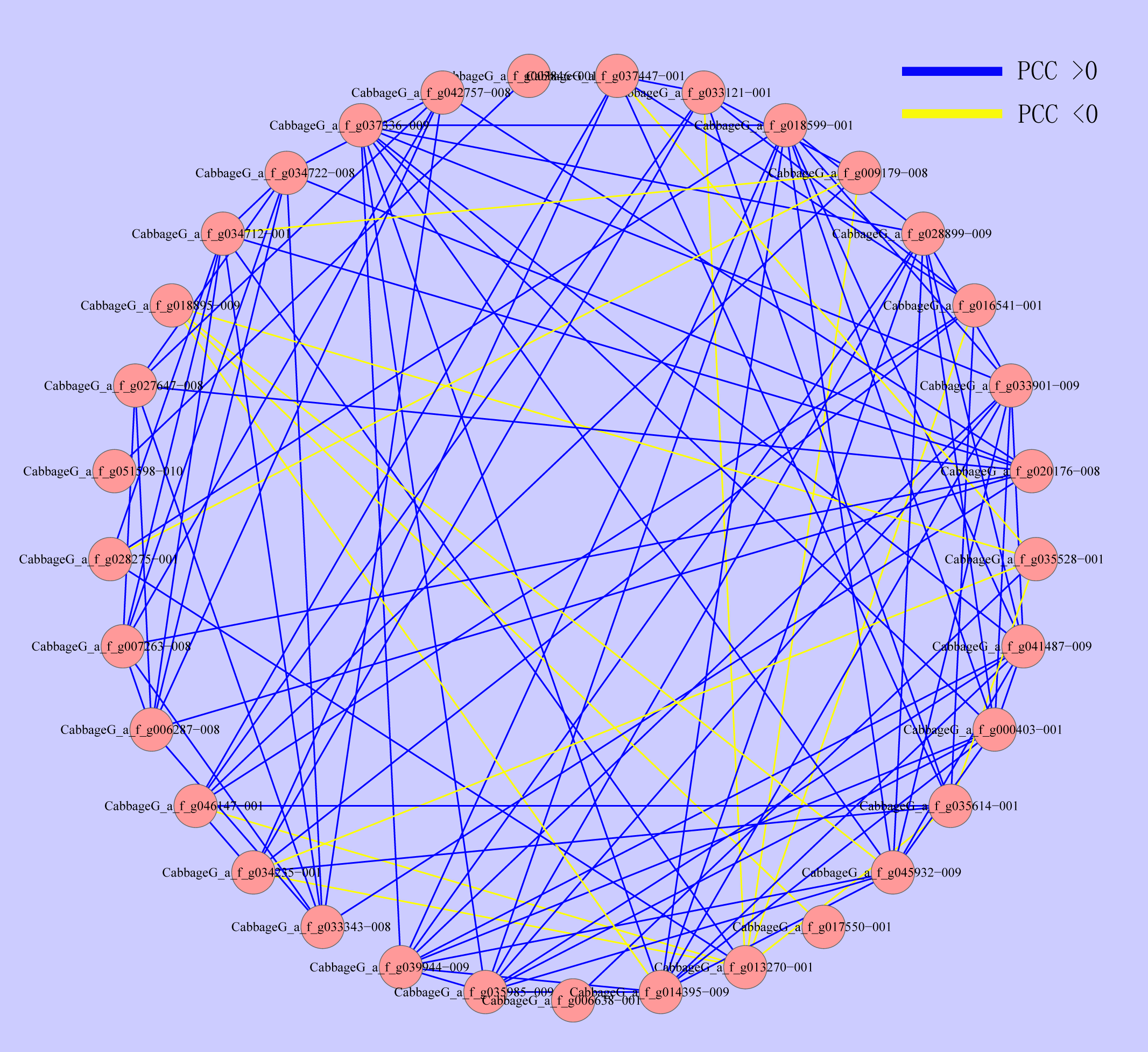

Supplement: Additional file 8: Figure S7. — The interaction network of variety-specific, differentially expressed, non-heading Chinese cabbage genes identified using a strict criterion. The Pearson’s correlation coefficients were calculated according to the transcriptome data of the five NHCC varieties. [file 12864_2015_1534_MOESM8_ESM.png]

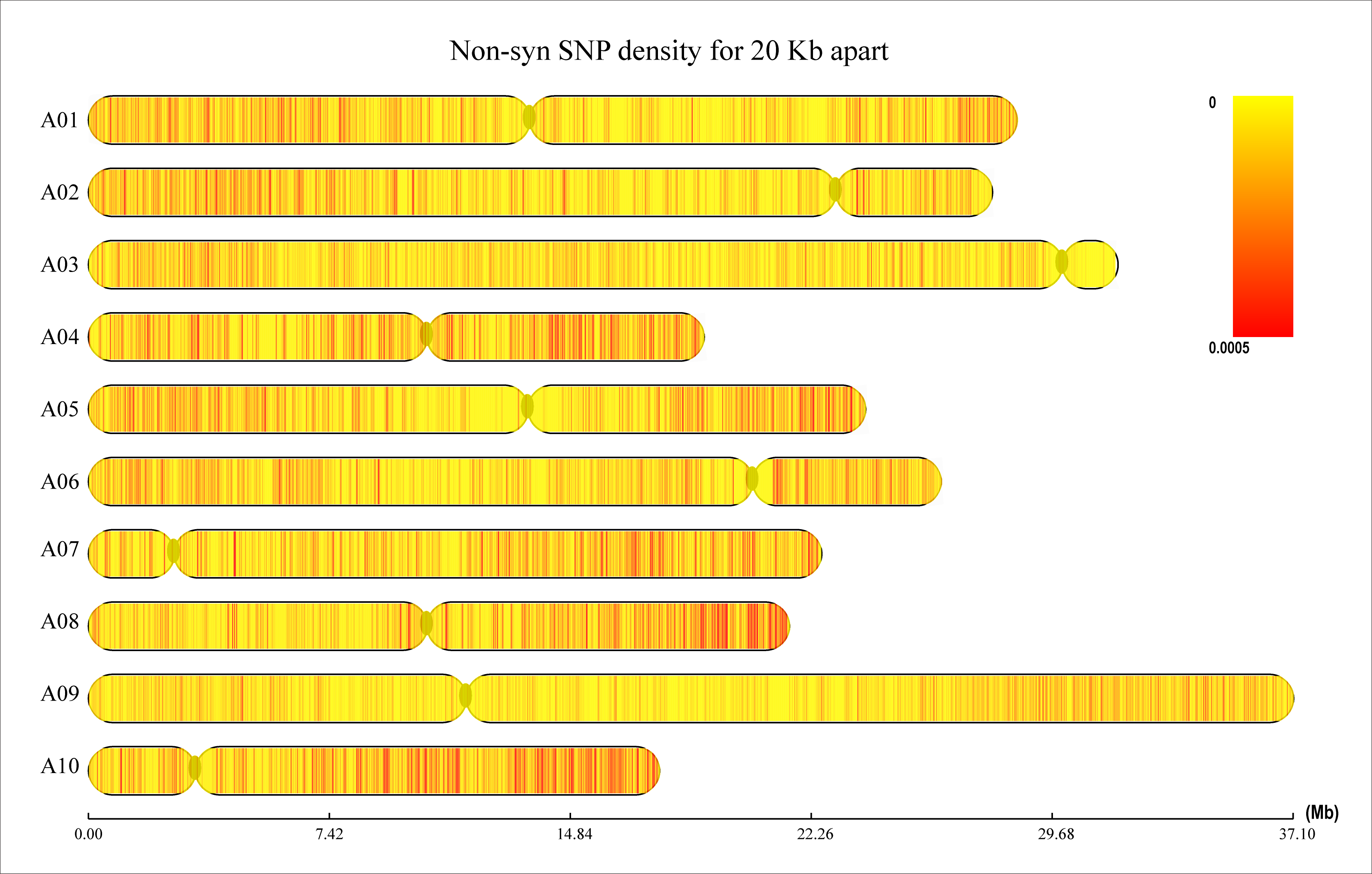

Supplement: Additional file 9: Figure S8. — The density of non-synonymous SNPs in non-heading Chinese cabbage accession on 10 chromosomes. The number of non-synonymous SNPs was calculated using 20 Kb windows. [file 12864_2015_1534_MOESM9_ESM.png]
